# Suppression of wetting transition on evaporative fakir droplets by using slippery superhydrophobic surfaces with low depinning force

**DOI:** 10.1038/s41598-023-29163-1

**Published:** 2023-02-09

**Authors:** Jubair A. Shamim, Yukinari Takahashi, Anjan Goswami, Nadeem Shaukat, Wei-Lun Hsu, Junho Choi, Hirofumi Daiguji

**Affiliations:** 1 Department of Mechanical Engineering, The University of Tokyo, 7-3-1 Hongo, Bunkyo-Ku, Tokyo, 113-8656 Japan; 2 Department of Mechanical Engineering, Imperial College London, London, SW7 2AZ UK; 3 Center for Mathematical Sciences, Pakistan Institute of Engineering and Applied Sciences, Nilore, 45650 Islamabad Pakistan

**Keywords:** Engineering, Mechanical engineering

## Abstract

This study experimentally investigated the evaporation and wetting transition behavior of fakir droplets on five different microstructured surfaces. Diamond-like carbon was introduced as the substrate, and the influence of varying the width, height, and pitch of the micropillars was assessed. The experimental results showed that the interfacial properties of the surfaces change the evaporation behavior and the starting point of the wetting transition. An important result of this study is the demonstration of a slippery superhydrophobic surface with low depinning force that suppresses the transition from the Cassie–Baxter state to the Wenzel state for microdroplets less than 0.37 mm in diameter, without employing large pillar height or multiscale roughness. By selecting an appropriate pillar pitch and employing tapered micropillars with small pillar widths, the solid–liquid contact at the three-phase contact line was reduced and low depinning forces were obtained. The underlying mechanism by which slippery superhydrophobic surfaces suppress wetting transitions is also discussed. The accuracy of the theoretical models for predicting the critical transition parameters was assessed, and a numerical model was developed in the surface evolver to compute the penetration of the droplet bottom meniscus within the micropillars.

## Introduction

**Figure 1:**
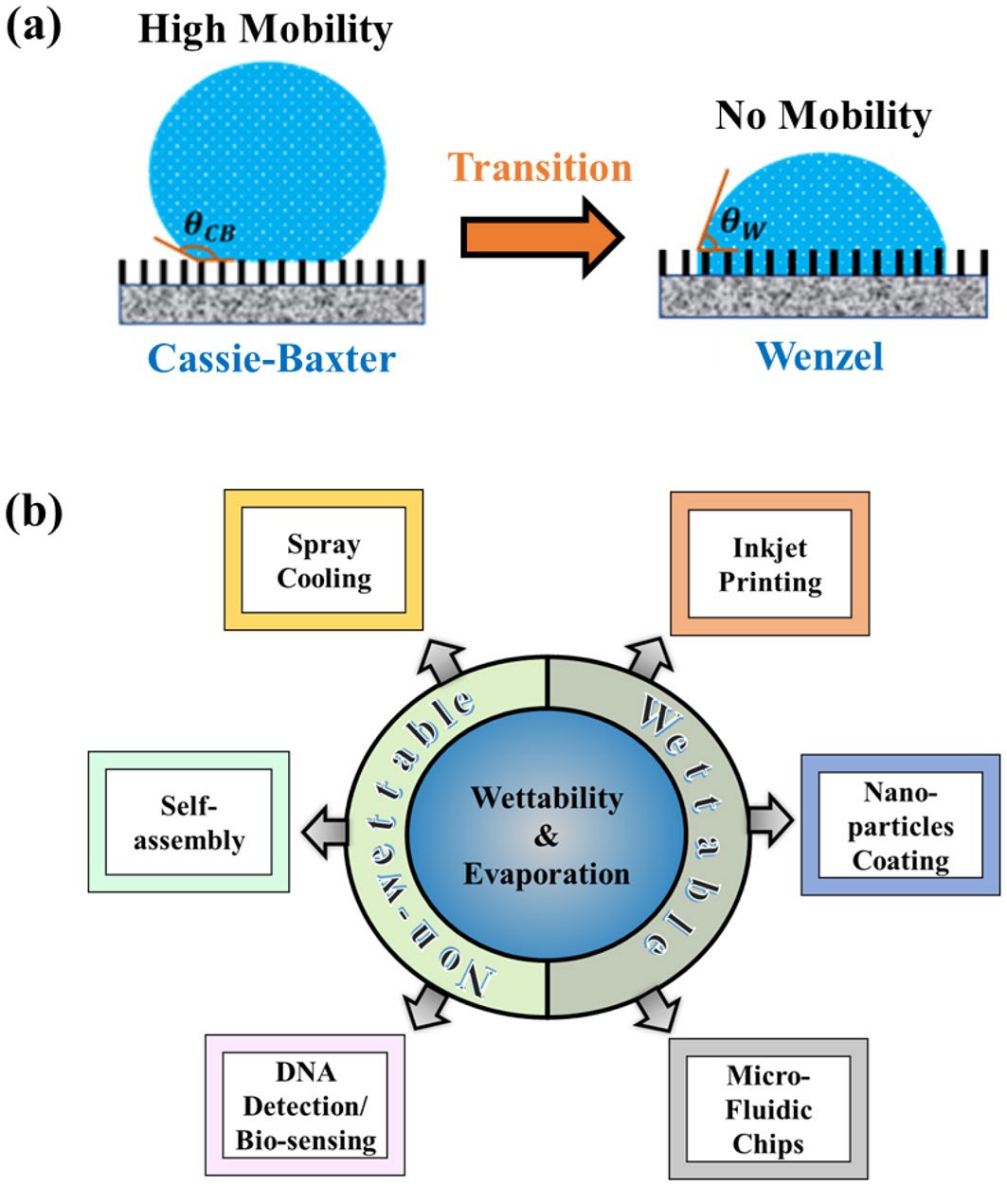
(**a**) Schematic of the Cassie–Baxter to Wenzel transition and (**b**) application of surface wettability and evaporation.

When a small millimeter-sized water droplet is placed on a superhydrophobic surface (SHS) comprising micron-sized pillars, it initially maintains a quasi-spherical shape and exhibits a large apparent contact angle (CA). This droplet is also known as the “fakir droplet”^1^ and is said to be in the Cassie–Baxter (CB) state with high mobility. Under the influence of external perturbations (e.g., evaporation^2^, vibration^3^, acoustic wave^4^, and electrical voltage^5^), the liquid invades the micropillars, and a fakir droplet undergoes a wetting transition from the CB to the Wenzel state, as shown in Fig. 1a. As a result, the droplet loses its mobility, and the surface becomes wettable. Surface wettability and droplet evaporation are key to many engineering applications, including spray cooling^6^, self-assembly^7^, DNA detection^8^, biosensing^9^, inkjet printing^10^, nanoparticle coating^11^, and microfluidic chip design^12^, as shown in Fig. 1b. Owing to this widespread implementation, understanding the physical mechanism of wetting phenomena has become a topic of fundamental interest^13,14^.

**Figure 2:**
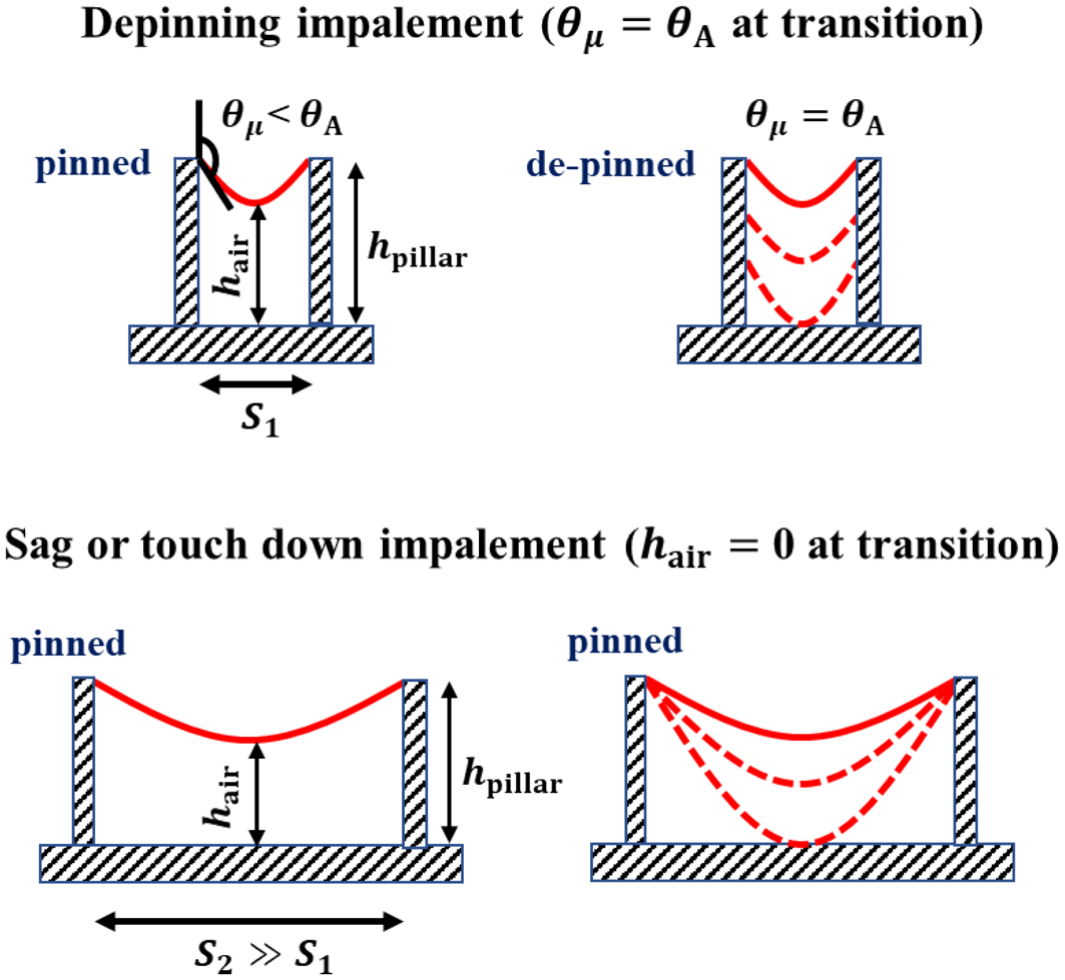
Mechanism of wetting transition on micropillared surfaces.

To date, two fundamental mechanisms, namely, “depinning impalement” and “sag or touch down impalement”, have been proposed to elucidate the wetting transition of evaporating droplets on microtextured surfaces, as shown in Fig. 2^15^. When the interpillar spacing is very small, the penetration of the droplet bottom meniscus within the micropillars is negligible. During evaporation, when the CA between the liquid–air interface and micropillars (${\theta }_{\mu }$) reaches the advancing CA (${\theta }_{\mathrm{A}}$), the three-phase contact line (TPCL) depins and vertically slides along the micropillar sidewall, and thus, the transition occurs. In contrast, for a sufficiently large interpillar spacing, the TPCL remains pinned with the micropillar sidewall, and the deflection of the droplet bottom meniscus becomes significant with increasing Laplace pressure during evaporation. The transition occurs when the meniscus touches the bottom of the substrate.

Several studies investigated the wetting and evaporation phenomena of fakir droplets on various micropatterned solid surfaces^2,16–21^. Bormashenko^22^ reviewed the advances in understanding the wetting transition phenomena on micropatterned surfaces. Studies on the modeling and simulation^23–27^, direct imaging^15,19,28–30^, and analytical techniques^31–33^ are also abundant in the literature. Impact dynamics of wetting transitions on micropillar surfaces were also investigated. Liu et al.^34^ found that in sub-millimeter scale tapered micro/nano textures, the capillary energy stored in the penetrating liquid is converted into upward motion, causing the droplet to jump without retracting, unlike conventional droplet repulsion^35^. They attributed this to the fact that on straight micropillars the stored capillary energy is not converted to upward motion because the transition from CB state to Wenzel state is more likely to occur. Later, Bro et al.^36^ reproduced the microscopic pancake bounce on a macro scale.

The stability of the CB state is key to the functionality of superhydrophobic surfaces in real-world applications^37^. To date, several efforts^38–41^ have been made to suppress the wetting transition. However, those studies employed liquid repellant surfaces with complex microstructures, such as microposts with doubly re-entrant nano-overhangs^39^, mushroom-double re-entrant fibril arrays^40^, or salvinia-like slippery surfaces^41^, which enhance the fabrication difficulty. The existing literature^42–44^ also reiterates that a hierarchical nanostructure is crucial for maintaining a stable CB state owing to the effect of air entrapment below the droplet. However, such dual-scale nanostructures are fragile and can be easily degraded under real operating conditions. Hence, a complex armored design^45^ was suggested to protect the nanostructure, thus increasing the fabrication difficulty. Thus, demonstrating a robust SHS with simple micropillars on which very small micron-sized droplets can stably maintain the CB state remains challenging.

In addition, several studies proposed the essential criteria of solid surfaces to prevent the collapse of the CB state. Zhang et al.^46^ showed that reducing the solid–liquid interaction or coating microstructure with nanostructures favored the reverse Wenzel to CB state transition for microdroplets. Zheng and Zhao^47^ presented the model for critical hydraulic pressure based on the area fraction to avoid the CB to Wenzel state transition. They hypothesized that reducing the pillar width and spacing to the nanometer scale would result in very high critical pressure for transition comparable to the impact pressure of the raindrop. Zhang et al.^24^ proposed the theory of critical height to obtain a stable CB state. On a microtextured surface with pillars of diameter 3 µm and mutual distance 17 µm, Reyssat et al.^17^ showed that when pillar height increased from 4.8 µm to 36.5 µm, liquid penetration could be avoided. Park et al.^48^ proposed the idea of manipulating the spacing-to-diameter ratio (*s*/*d*) and height-to-diameter ratio (*h*/*d*) to maintain the CB state. Whyman and Bormashenko^49^ showed that increasing the energy barrier using multiscale roughness could maintain the CB state.

From the above literature survey, it is clear that previous studies have focused primarily on increasing the micropillar height and critical pressure, manipulating *s*/*d* and* h*/*d* ratios, and using multiscale roughness to maintain the CB state. To date, the suppression of the CB to Wenzel state transition by employing a slippery SHS with low depinning force, ${F}_{\mathrm{D}}$, i.e., the force required for the depinning of the TPCL at the top of the micropillar, is not yet well understood. Based on the results of a previous study^50^, this study proves that a slippery SHS with significantly low ${F}_{\mathrm{D}}$ (~ 0.80 mN/m) can suppress the CB to Wenzel state transition without employing multiscale roughness or large pillar height. Low ${F}_{\mathrm{D}}$ was obtained by reducing the solid–liquid contact in the TPCL. The reduction of solid–liquid contact was achieved by (i) selecting an appropriate pillar pitch that reduces the normalized TPCL, $\delta$, i.e., ratio of pillar perimeter to pitch, below 0.5 and (ii) employing tapered micropillars with a small pillar width of 1.87 µm at the top end.

In this study, it is demonstrated the simple, robust, and slippery SHS with tapered micropillars with the actual height of 6.72 µm, unlike in the previous study^17^ that assumed a pillar height of 36.5 µm, is resistant to liquid penetration during evaporation, even for microdroplets (droplet diameter: < 0.37 mm). Furthermore, previous studies^18,51–53^ have reported that the emergence of a characteristic evaporation mode during the wetting transition of water droplets on SHS, which depended on the nature of the solid–liquid interface. Therefore, in this study, the influence of slippery interfaces on the evaporation dynamics of fakir droplet is also investigated. The accuracy of existing theoretical models for predicting critical transition parameters at new surfaces is also discussed. Finally, a numerical model is proposed to predict the meniscus penetration of a droplet suspended on a unit cell consisting of four micropillars, based on Young’s CA^54^.

## Method

### Fabrication of DLC-based SHS

**Figure 3:**
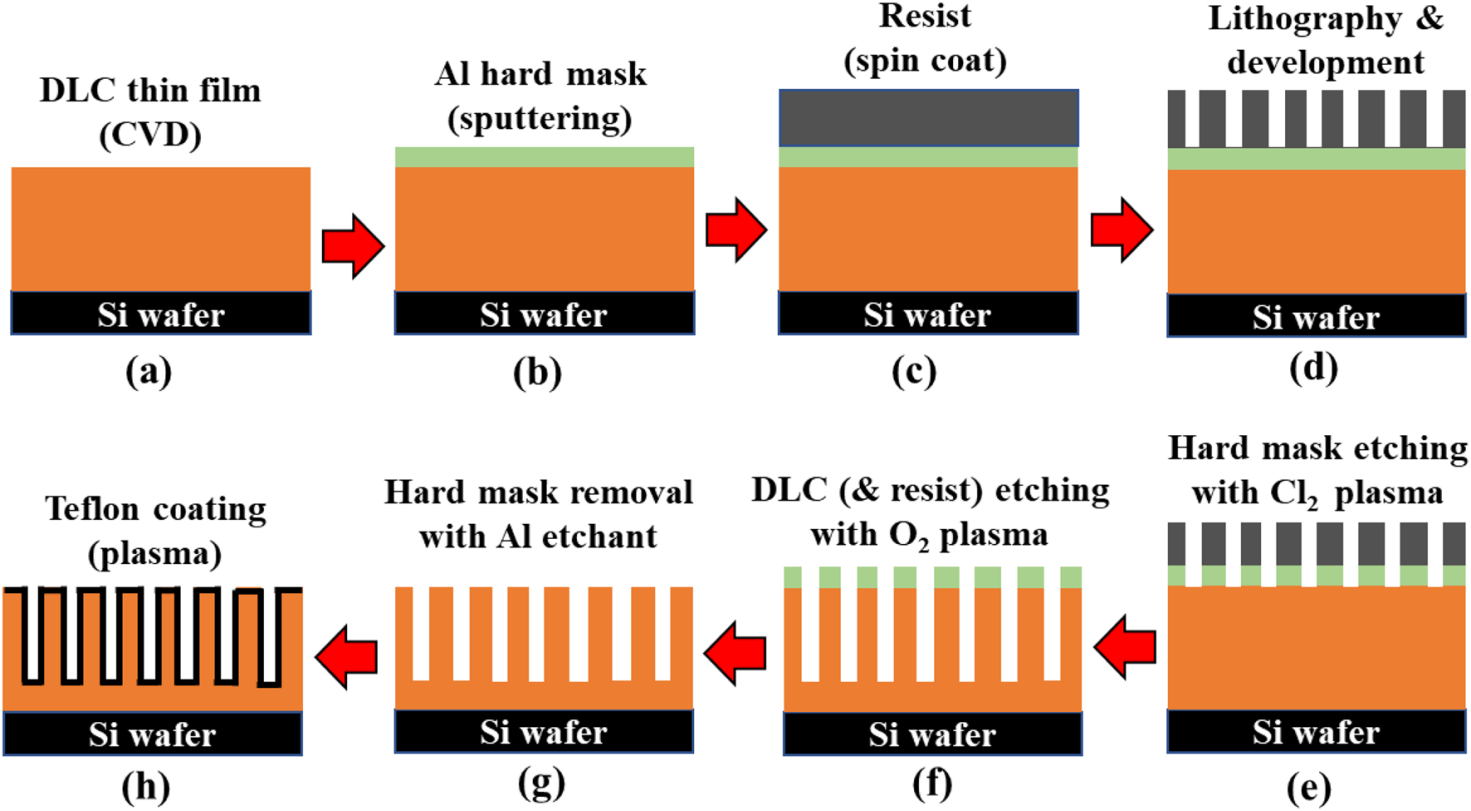
Schematic of process steps to fabricate DLC-based micropillared surfaces.

Schematics of the surface fabrication steps are shown in Fig. 3. The surfaces were designated *w*3-*p*9-*h*3, *w*3-*p*9-*h*6, *w*3-*p*15-*h*3, *w*3-*p*15-*h*6, and *w*25-*p*75-*h*8, according to the design width (*w*), pitch (*p*) (center-to-center distance), and height (*h*) of the micropillars. Further details of the surface fabrication are provided in Supplementary Note 1. The conditions for reactive ion etching (RIE) using O_2_ plasma in the Diamond-like carbon (DLC) layer are summarized in Supplementary Table S1.

However, after fabrication, the actual micropillar dimensions varied slightly from the design dimensions, as measured by scanning electron microscopy (SEM), as shown in Supplementary Fig. S1. Detailed SEM images of the micropillar arrays on different surfaces are shown in Supplementary Figs. S2 and S3. The actual micropillar dimensions of the different surfaces are summarized in Supplementary Table S2. Furthermore, the surfaces were characterized in terms of the static CA, roll-off angle (ROA), advancing CA, receding CA, and hysteresis (CAH). The measured values are listed in Supplementary Table S3. All details regarding the characterization of the fabricated surfaces are described in Supplementary Note 2.

DLC was introduced as a new SHS substrate owing to its excellent wear resistance, low friction coefficient, high hardness, and chemical inertness^55^. DLC has the potential to design mechanically robust SHS for practical applications^56^. Durability test data, thermal stability, chemical inertness, and abrasion resistance of *w*3-*p*9-*h*6 surface representing DLC micropillars are shown in Supplementary Note 3. Supplementary Figs. S4 and S5 show the static CA, ROA, and CAH obtained during thermal stability, chemical inertness, and abrasion resistance experiments. Supplementary Fig. S6 shows the optical microscope image of the *w*3-*p*9-*h*6 surface at the end of the chemical inertness experiment. The above test data show that the Teflon-coated DLC micropillars were not damaged when heated to 105 °C and immersed in 98% H_2_SO_4_. They also showed wear resistance of 20 cm under a stress of 2.18 kPa with #1000 grit sandpaper. The use of DLC micropillars in this study ensured good reproducibility and accuracy of the experimental data over a long period of time. However, further improvement of the wear resistance is beyond the scope of this paper and is a subject for future study.

### Experimentation of wetting transition

**Figure 4:**
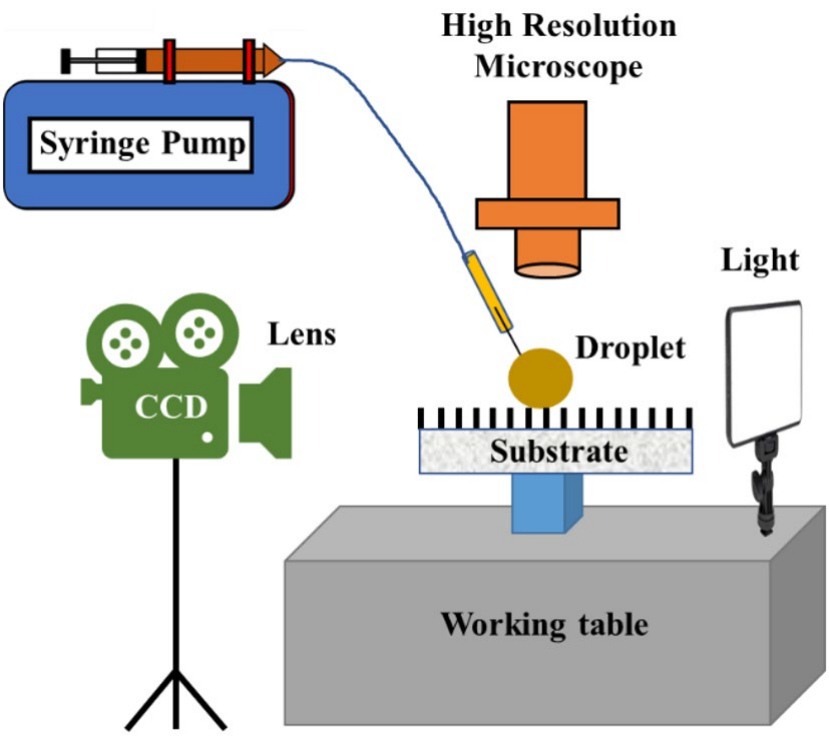
Schematic of the experimental setup for the wetting transition experiment.

A state-of-the-art experimental facility (Fig. 4) was assembled at the University of Tokyo to perform wetting transition experiments on DLC-based surfaces. The facility employed the latest sCMOS camera (Zyla 5.5, Andor-Oxford Instruments) to obtain the lateral view of the droplet and a high-resolution microscope (Olympus DSX 1000) to obtain the top view of the droplet during evaporation. The lateral and top views of the evaporating droplets were recorded at 10 and 1 fps, respectively. A real view of the experimental facility is shown in Supplementary Fig. S7.

First, a small droplet was generated using a syringe pump (Harvard Apparatus) and needle (Hamilton REF. 90031) connected via a Teflon tube and then gently placed on the surface. The initial droplet diameter and volume at the beginning of the wetting experiment for each surface are listed in Supplementary Table S4. Then, the droplet was left to evaporate under room conditions [20–40% relative humidity and 20–25 °C, measured by a humidity sensor (Rotronic HC2A-S)], and images were recorded from the side and top. An in-house image-processing code was developed in MATLAB to obtain the CA, contact length (CL) of the droplet bottom with the surface, and droplet diameter from the recorded data. Surface *w*3-*p*15-*h*6 was chosen to plot the uncertainty of the experimental measurements as this surface demonstrated the best performance. From Supplementary Fig. S8, the experimental results of the time course of CA could be reproduced within a deviation of ± 3.5%.

## Results

### Wetting transition process

**Figure 5:**
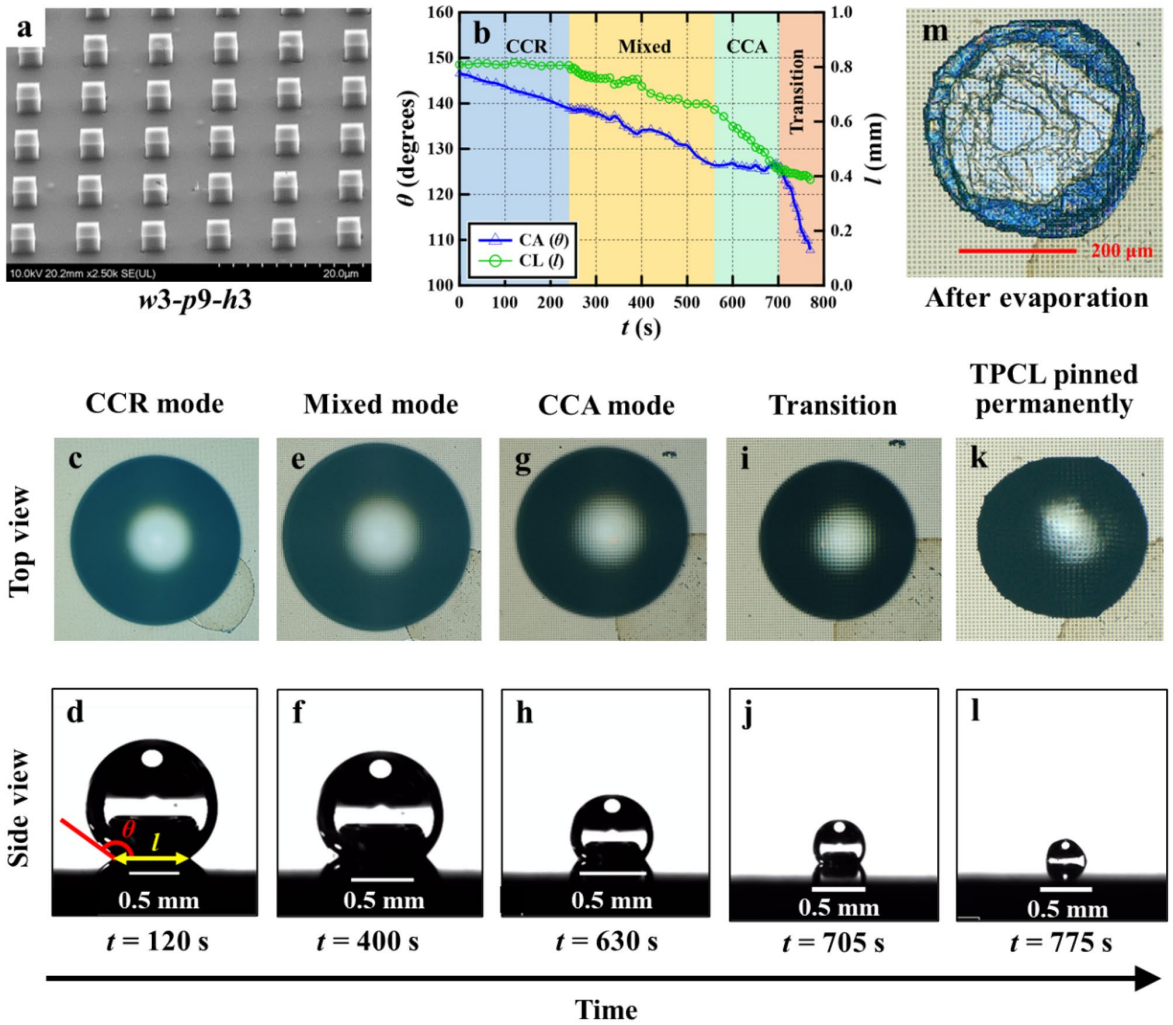
Illustration of the wetting transition process on surface *w*3-*p*9-*h*3.

First, we elucidated the process of wetting transition on the baseline surface *w*3-*p*9-*h*3 (Fig. 5a). The time course of CA and CL ($\theta -t$ and $l-t$ curves) during droplet evaporation on this surface is shown in Fig. 5b.

From Fig. 5b, one can see that until the first 245 s, the CL did not change with time as the TPCL remained pinned to the micropillar edges, and CA decreased from 146.6° to 138.9° (i.e., 1.9 degrees/min). Hence, it is called the constant contact radius (CCR) mode of evaporation. During this period, the spherical droplet sat on top of the micropillars in the CB state. Figure 5c and d respectively represent the snapshot of top and lateral views of droplet evaporating in CCR mode at *t* = 120 s. From 245 to 560 s, the CL and CA (2.4 degrees/min) decreased with time (mixed mode of evaporation). During this period, a moving TPCL appeared due to the local pinning and depinning with the pillar edges. Figure 5e and f respectively represent the snapshot of top and lateral views of droplet evaporating in mixed mode at *t* = 400 s. From 560 to 705 s, the droplet evaporated in constant contact angle (CCA) mode, i.e., the CL decreased with time, but the CA remained unchanged with time. Still, the TPCL showed local pinning and depinning with the pillar edges. Figure 5g and h respectively represent the snapshot of top and lateral views of droplet evaporating in the CCA mode at *t* = 630 s.

Finally, at *t* = 705 s, liquid penetration occurred with a rapid decrease in the CA, known as the transition from the CB to the Wenzel state. However, all the points on TPCL could still depin from the pillar edges (Fig. 5i,j). At *t* = 775 s, TPCL permanently pinned to the micropillars again, and the droplet volume became much smaller due to the evaporation. At this moment, the effect of pinning became evident (Fig. 5k,l), and finger-like spikes were formed at the droplet periphery (magnified view in Supplementary Fig. S9). Figure 5m shows traces of dust particles (trapped under the droplet) remaining on the surface after evaporation. Supplementary Video 1 shows permanent pinning of TPCL and completion of droplet evaporation on *w*3-*p*9-*h*3 surface. Reyssat et al.^17^ also reported a similar image of the stain resulting from the evaporation-driven flow of dust in the water. Detailed pictures of the wetted area after complete evaporation of the droplet on the five surfaces are shown in Supplementary Fig. S10.

**Figure 6:**
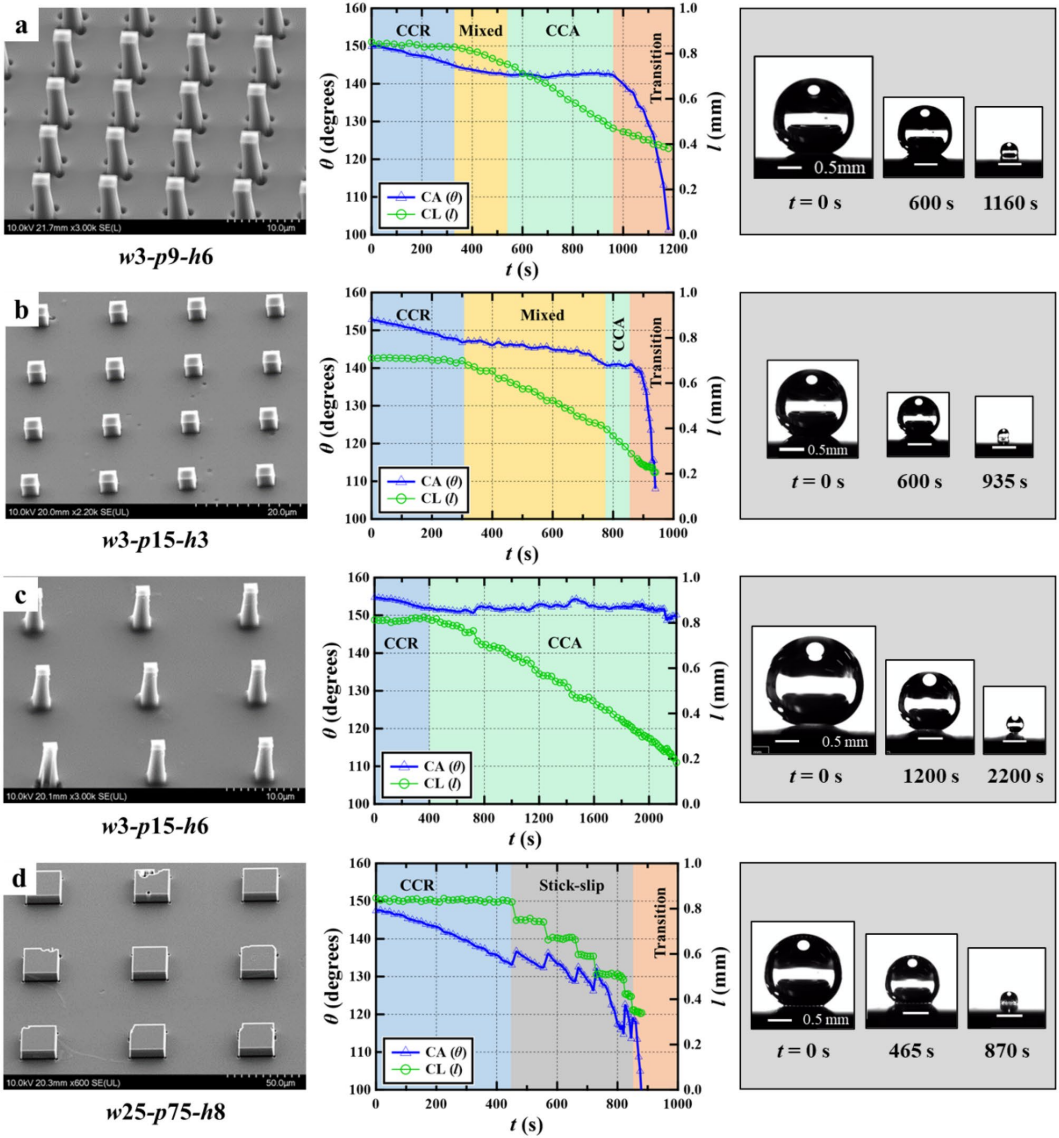
Dynamics of evaporation on the other four different DLC-based surfaces.

Next, we discuss the evaporation dynamics and appearance of distinct evaporation modes during the transition process on all five surfaces. As shown in Figs. 5 and 6a–d, the droplets were initially in the CB state with a high apparent CA, and evaporation was initiated in a CCR mode on all five surfaces. The TPCL was pinned to the micropillars, and CL did not change, but CA decreased due to the loss of droplet volume during evaporation^52^. The appearance of the CCR mode at the onset of evaporation was also reported in another study^53^.

Following the CCR mode, a mixed evaporation mode appeared on the *w*3-*p*9-*h*3 (Fig. 5), *w*3-*p*9-*h*6 (Fig. 6a), and *w*3-*p*15-*h*3 (Fig. 6b) surfaces. After the mixed mode, the CCA mode also appeared on these three surfaces prior to the beginning of the transition period. The duration of the various evaporation modes mentioned above and the trends of the CA over time during each evaporation mode depended on the unique interfacial characteristics of each surface. For example, the mixed mode was the smallest on the *w*3-*p*9-*h*6 surface, while the CCA mode was the smallest on the *w*3-*p*15-*h*3 surface. The mixed mode also shows a very slow decrease in CA for the *w*3-*p*9-*h*6 surface (0.5°/min) and *w*3-*p*15-*h*3 surface (0.8°/min), compared to the *w*3-*p*9-*h*3 surface (2.4°/min). Consequently, the CA was above 140° at the *w*3-*p*9-*h*6 and *w*3-*p*15-*h*3 surfaces, while decreased to 126.1° at the *w*3-*p*9-*h*3 surface before the start of the transition.

On the *w*3-*p*15-*h*6 surface (Fig. 6c), evaporation switched from the initial CCR to the CCA mode. Unlike on previous surfaces, here, the transition did not occur, and the CA remained close to 150° until the measurable range of the droplet diameter, i.e., 0.37 mm. Finally, the evaporation on the *w*25-*p*75-*h*8 surface (Fig. 6d) switched from CCR to a distinct stick–slip mode^57^, i.e., the CL decreased in a stepwise manner, and the CA repeatedly increased and decreased until the transition started. The CA before the start of the transition was ~ 118°. The appearance of the above distinct evaporation modes on the five surfaces in this study might be subject to the different magnitudes of the depinning force on these surfaces (Supplementary Table S5 and Supplementary Fig. S11a).

## Discussion

### Evaluation of existing wetting transition models

In this section, we discuss the applicability of existing wetting transition models from the literature to predict the critical parameters during transition for the five surfaces covered in this study. For the theoretical critical pressure at transition (${\Delta p}_{\mathrm{c},\mathrm{th}}$), the following equation was employed^47,58^:

1 $${\Delta p}_{\mathrm{c},\mathrm{ th}}=-\frac{4 {\sigma }_{\mathrm{lg} }w\mathrm{ cos}{\theta }_{\mathrm{A}}}{{p}^{2}-{w}^{2}}$$

where ${\sigma }_{\mathrm{lg}}$ is the surface tension at the liquid–gas interface of the droplet, $w$ is the actual width of the square micropillars measured by SEM (Supplementary Table S2), ${\theta }_{\mathrm{A}}$ is the advancing CA on a flat surface (~ 115° for Teflon^59^), and $p$ is the actual pitch between micropillars. For the theoretical critical angle (${\theta }_{\mathrm{c},\mathrm{th}}$), the following equation was selected, which was derived based on the surface free energy^52^:

2 $${\theta }_{\mathrm{c},\mathrm{th}}={\mathrm{cos}}^{-1}\left(\frac{{f}_{\mathrm{s}}-1}{r-{f}_{\mathrm{s}}}\right)$$

where ${f}_{\mathrm{s}}$ is the solid fraction and $r$ is the roughness factor (Supplementary Table S5).

The experimental wetting pressure (${\Delta p}_{\mathrm{w},\mathrm{ exp}}$) was approximated as the Laplace pressure at the liquid–air interface, according to the following equation^60^:

3 $${\Delta p}_{\mathrm{w},\mathrm{ exp}}= \frac{2{\sigma }_{\mathrm{lg} }}{{R}_{\mathrm{c},\mathrm{exp}}}$$

where ${R}_{\mathrm{c},\mathrm{exp}}$ is the critical droplet radius at transition from the experiment.

The experimental critical angle (${\theta }_{\mathrm{c},\mathrm{exp}}$) was estimated from the image analysis at the onset of the transition. Table 1 shows a comparison between the theoretical predictions and experimental values of the above critical parameters. The experimental wetting pressure and critical angle for the *w*3-*p*15-*h*6 surface are not reported in Table 1 because no transition was observed up to a droplet diameter of 0.37 mm. From Table 1, it is evident that Eq. (1) highly overpredicted the critical pressure for the first two surfaces with a smaller pitch (9 µm). The deviation decreased for the *w*3-*p*15-*h*3 surface as the pitch increased from 9 to 15 µm and was minimal for the *w*25-*p*75-*h*8 surface having the largest pitch (75 µm). On the other hand, Eq. (2) underestimated the ${\theta }_{\mathrm{c},\mathrm{exp}}$ for the *w*3-*p*9-*h*6 surface and overestimated it for all other surfaces. Nevertheless, the deviations were reasonable for the first three surfaces but were largest for the *w*25-*p*75-*h*8 surface. Thus, neither theoretical model could precisely capture the critical transition parameters of this work.

**Table 1:** Comparison of critical parameters during the transition.

Surface	Theoretical		Experimental	
	${\Delta p}_{\mathrm{c},\mathrm{th}}$ (Pa)	${\theta }_{\mathrm{c},\mathrm{th}}$ (degree)	${\Delta p}_{\mathrm{w},\mathrm{exp}}$ (Pa)	${\theta }_{\mathrm{c},\mathrm{exp}}$ (degree)
*w*3-*p*9-*h*3	5.01 × 10^3^	132.4	5.64 × 10^2^	125.6
*w*3-*p*9-*h*6	3.44 × 10^3^	124.4	4.17 × 10^2^	140.0
*w*3-*p*15-*h*3	1.65 × 10^3^	149.2	7.38 × 10^2^	139.4
*w*3-*p*15-*h*6	9.97 × 10^2^	145.0	–	–
*w*25-*p*75-*h*8	5.66 × 10^2^	149.6	8.00 × 10^2^	118.0

The key reason behind the high discrepancy between ${\Delta p}_{\mathrm{w},\mathrm{ exp}}$ and ${\Delta p}_{\mathrm{c},\mathrm{ th}}$ for surfaces with small pitch might be as follows: Eq. (1) was originally derived from the vertical force balance between hydraulic pressure and surface tension along the micropillar walls acting on the interpillar floating water column of a droplet in equilibrium with pinned TPCL^47^. However, for an evaporating droplet on surfaces with small pitch under this study, a moving TPCL appeared with alternate pinning and depinning. During the movement of TPCL, the Laplace pressure continued to increase due to evaporation. Therefore, in the experiment, transition occurred at a much lower pressure than ${\Delta p}_{\mathrm{c},\mathrm{ th}}$. This situation was not accounted for in Eq. (1).

### Evaluation of suspended meniscus penetration

**Figure 7:**
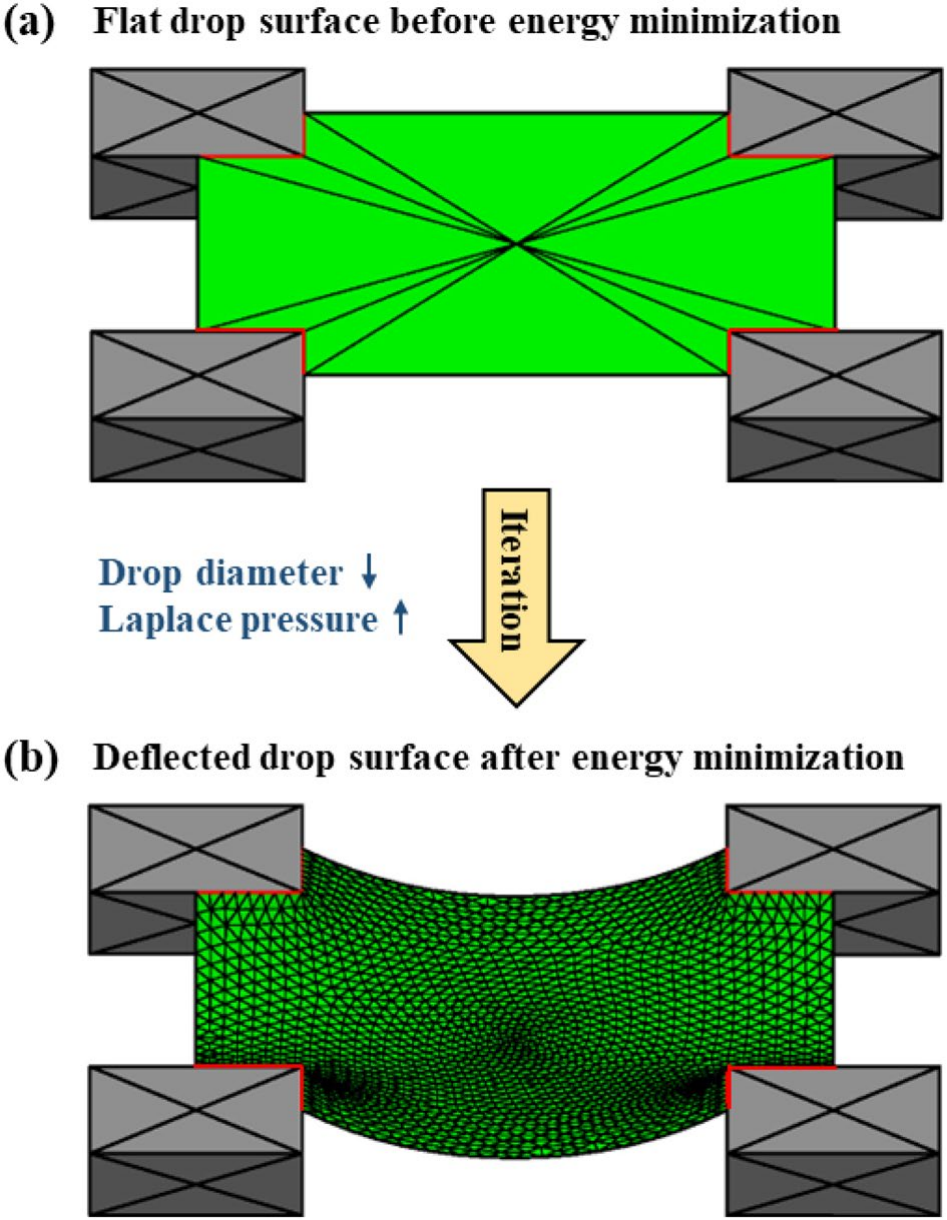
(**a**) Planar liquid surface in the SE model on a unit cell and (**b**) the deflected surface after energy minimization.

In this section, a finite-element-based open source code *Surface Evolver* (SE)^61^ was used to numerically approximate the penetration of the meniscus of the droplet suspended on a unit cell under different Laplace pressures. The governing equations of the SE model are described in Supplementary Note 6. First, the bottom of the droplet was defined as a planar liquid surface whose edges were pinned to a unit cell comprising four micropillars (Fig. 7a). The final equilibrium shape (Fig. 7b) was achieved by successive refinement of the triangular meshes and iterative energy-minimizing steps. The iterations were repeated until the energy change of the evolved surface was < 10^–8^ erg. The numerical penetration (${\Delta }_{\mathrm{SE}}$) was defined as the vertical distance from the pillar top to the minimum point of the evolved equilibrium surface owing to the energy minimization (Supplementary Fig. S12).

**Figure 8:**
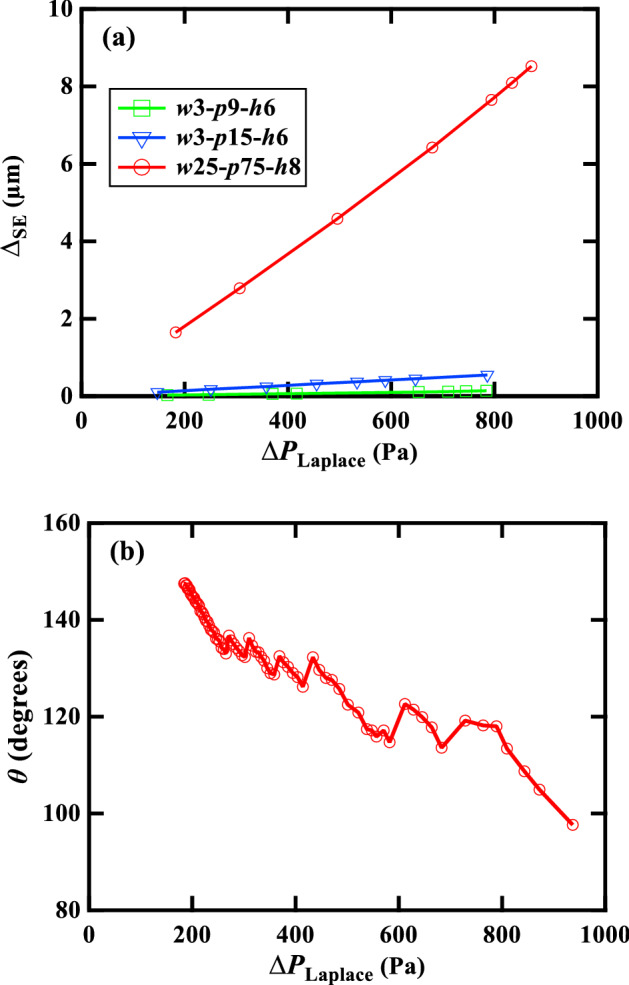
(**a**) Deflection of the droplet bottom meniscus vs. Laplace pressure (${\Delta }_{\mathrm{SE}}-{\Delta P}_{\mathrm{Laplace}}$ curve) numerically obtained in the SE model and (**b**) experimentally obtained CA vs. Laplace pressure ($\theta -{\Delta P}_{\mathrm{Laplace}}$ curve) for the *w*25-*p*75-*h*8 surface.

The calculated deflection of the droplet bottom meniscus vs. Laplace pressure (${\Delta }_{\mathrm{SE}}-{\Delta P}_{\mathrm{Laplace}}$ curve) for the three different surfaces are shown in Fig. 8a. The *w*3-*p*9-*h*6, *w*3-*p*15-*h*6, and *w*25-*p*75-*h*8 surfaces were selected as representative surfaces with varying interpillar distances and micropillar widths. When the interpillar spacing was the largest (*w*25-*p*75-*h*8), sagging became dominant, and the penetration increased significantly with increasing ${\Delta P}_{\mathrm{Laplace}}$. On the *w*25-*p*75-*h*8 surface, ${\Delta }_{\mathrm{SE}}$ was 8.52 µm at ${\Delta P}_{\mathrm{Laplace}}$ = 871 Pa, which was almost equal to the actual micropillar height (8.53 µm). Hence, it can be inferred that the suspended meniscus touched the substrate bottom at this pressure, and the transition occurred.

An experimental plot of CA vs. Laplace pressure ($\theta -{\Delta P}_{\mathrm{Laplace}}$ curve) obtained for this surface is shown in Fig. 8b. At ${\Delta P}_{\mathrm{Laplace}}$ = 871 Pa, the corresponding $\theta$ ~ 104°, which was much smaller than the ${\theta }_{\mathrm{c},\mathrm{ exp}}$ of this surface (118.03°). Thus, the developed SE model could successfully predict the penetration of the suspended meniscus in the Wenzel state for a large interpillar distance. Conversely, in the case of a small interpillar distance (i.e., *w*3-*p*9-*h*6 and *w*3-*p*15-*h*6 surfaces), the increase in ${\Delta }_{\mathrm{SE}}$ was insignificant, even at a large ${\Delta P}_{\mathrm{Laplace}}$. Therefore, it can be said that the transition on these surfaces did not occur by the sagging of the bottom meniscus. Rather, the TPCL depinned and vertically slid along the pillars when ${\theta }_{\mu }$ reached ${\theta }_{\mathrm{A}}$ during evaporation.

Furthermore, the theoretical penetration (${\Delta }_{\mathrm{th}}$) was approximated by the following equation for comparison with the numerical penetration^31^:

4 $${\Delta }_{\mathrm{th}}=\frac{L}{2}\frac{1-\mathrm{sin}(\pi -{\theta }_{\mu })}{\mathrm{cos}(\pi -{\theta }_{\mu })} \left(=-\frac{\left(1-\mathrm{sin}{\theta }_{\mu }\right)L}{2\mathrm{cos}{\theta }_{\mu }}\right)$$

where $L$ is the interpillar spacing and ${\theta }_{\mu }$ is the CA with the micropillar, which can be approximated as follows for square pillars^31^:

5 $${\theta }_{\mu }={\mathrm{cos}}^{-1}\left(-{\Delta P}_{\mathrm{Laplace}}\frac{{p}^{2}-{w}^{2}}{4{w\sigma }_{\mathrm{lg}}}\right)$$

where $p$ is the pitch between pillars, $w$ is the width of the square pillar, ${\sigma }_{\mathrm{lg}}$ is the surface tension at the liquid–gas interface, and ${\Delta P}_{\mathrm{Laplace}}$ is the Laplace pressure at the liquid–air interface. A comparison between the numerical and theoretical penetrations of the above three surfaces at the experimental wetting pressure (for *w*3-*p*15-*h*6 surface Laplace pressure 778.37 Pa was used at 0.37 mm drop dia) is presented in Table 2. The theoretical model overestimated the penetration of the bottom meniscus for all three surfaces.

**Table 2:** Comparison of numerical and theoretical penetration of the bottom meniscus of the droplet.

Surface	${\Delta }_{\mathrm{th}}$ (µm)	${\Delta }_{\mathrm{SE}}$ (µm)
*w*3-*p*9-*h*6	0.09	0.07
*w*3-*p*15-*h*6	1.12	0.55
*w*25-*p*75-*h*8	8.45	7.71

### Proposed mechanism for the stability of the Cassie–Baxter state on slippery SHS

**Figure 9:**
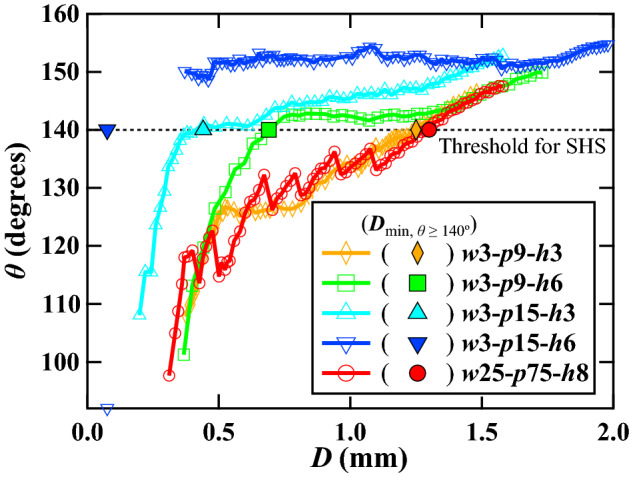
Plot of apparent CA vs. droplet diameter ($\theta -D$ curve) on five different surfaces.

To elucidate the stability of the CB state, a plot of CA vs. droplet diameter ($\theta -D$ curve) during evaporation on the five surfaces is shown in Fig. 9. The superiority of the five surfaces in terms of maintaining $\theta \ge 140^\circ$, i.e., the minimal droplet diameter that could maintain $\theta \ge 140^\circ$ (${D}_{\mathrm{min}, \theta \ge 140^\circ }$) for the five surfaces was in the following order from smallest to largest: *w*3-*p*15-*h*6 < *w*3-*p*15-*h*3 < *w*3-*p*9-*h*6 < *w*3-*p*9-*h*3 < *w*25-*p*75-*h*8. Here, we used $\theta =140^\circ$ as the reference value owing to the fact that one can consider the surface nearly superhydrophobic at $\theta =140^\circ$. The CAs on the *w*3-*p*9-*h*3 and *w*25-*p*75-*h*8 surfaces decreased below 140° for relatively larger droplet diameters ($D=1.25 \text{ mm}$ and $1.30 \text{ mm}$, respectively, for *w*3-*p*9-*h*3 and* w*25-*p*75-*h*8 surfaces) than those on the other three surfaces. However, the CAs on the *w*3-*p*9-*h*6 and *w*3-*p*15-*h*3 surfaces remained close to 140° until the droplet diameter reached 0.69 mm and 0.44 mm, respectively. Thus, the latter two surfaces demonstrated a better ability to maintain high CA until the droplet size became considerably small. However, on the *w*3-*p*15-*h*6 surface, the CA remained close to 150°, even when the droplet diameter reached 0.37 mm, yet no decreasing trend in CA was observed; thus, this surface outperformed all other surfaces. The CA could not be measured for smaller droplets with $D$ < 0.37 mm using the developed image processing code because the image quality deteriorated significantly. Supplementary Video 2 shows a comparison of the evaporation and transition behavior of the fakir droplet on the *w*3-*p*9-*h*3 and *w*3-*p*15-*h*6 surfaces.

Another comparison of the diameter of the wetted areas on the five surfaces after evaporation was completed is shown in Supplementary Fig. S10. From this figure, one can see that the diameter of the wetted area on the best-performing *w*3-*p*15-*h*6 surface was only 75 µm, which was approximately five times smaller than that of the baseline *w*3-*p*9-*h*3 surface. Thus, for simplicity, we assumed that the CA on the *w*3-*p*15-*h*6 surface was maintained above 140° until the droplet diameter reached 75 µm.

The above observations can be explained as follows: the stability of the CB state primarily depends on the depinning ability of the TPCL^37^. The force required for TPCL depinning becomes significantly smaller with a decrease in the solid–liquid contact area. A small pillar width and appropriately larger pitches result in small solid fractions (${f}_{\mathrm{s}}$) and shorter normalized TPCLs, $\delta$, respectively^50^. Consequently, the depinning force, ${F}_{\mathrm{D}}$, would be significantly reduced^50^ and a rapidly moving TPCL would appear. More details on the mechanism of wetting transition suppression on slippery SHS, including an illustration (Supplementary Fig. S13), are given in Supplementary Note 7. The approximated values of ${f}_{\mathrm{s}}$, $\delta$, and ${F}_{\mathrm{D}}$ for the actual pillar dimensions for all five surfaces are shown in Supplementary Table S5. Supplementary Fig. S11b shows the plot of ${F}_{\mathrm{D}}$ vs. $\delta$ for five surfaces covered this study.

Supplementary Table S5 shows that the ${F}_{\mathrm{D}}$ of the five surfaces under this study decreases in the following order: *w*25-*p*75-*h*8 > *w*3-*p*9-*h*3 > *w*3-*p*9-*h*6 > *w*3-*p*15-*h*3 > *w*3-*p*15-*h*6. This contrasts with the previously mentioned order for maintaining $\theta \ge 140^\circ$. Thus, it can be inferred that reducing the depinning force was the key to maintaining high CA. Among the five surfaces, *w*3-*p*15-*h*6 had the lowest actual solid fraction (${f}_{\mathrm{s},\mathrm{a}}$ = 1.51%, Supplementary Table S5), resulting in a very low ${F}_{\mathrm{D}}$ (0.80 mN/m) on this surface. Supplementary Table S2 shows that the actual micropillar width was the smallest (*w* = 1.87 µm) for this surface. During fabrication, RIE on the *w*3-*p*15-*h*6 surface was continued for a longer duration to achieve a 6 µm pillar height. The top of the micropillars was etched faster than the bottom, which led to the formation of conical micropillars (Supplementary Fig. S3d), resulting in the smallest micropillar width. In addition, the interpillar spacing of the *w*3-*p*15-*h*6 surface was sufficiently large to significantly reduce $\delta$ below 0.5 ($\delta$ = 0.49, Supplementary Table S4). Owing to this combined effect, a nearly super-slippery surface with a very small ${F}_{\mathrm{D}}$ was obtained. As a result, a high CA at small droplet diameter ($\theta \approx 150^\circ$ at $D=0.37\mathrm{ mm}$) could be maintained on the *w*3-*p*15-*h*6 surface.

**Figure 10:**
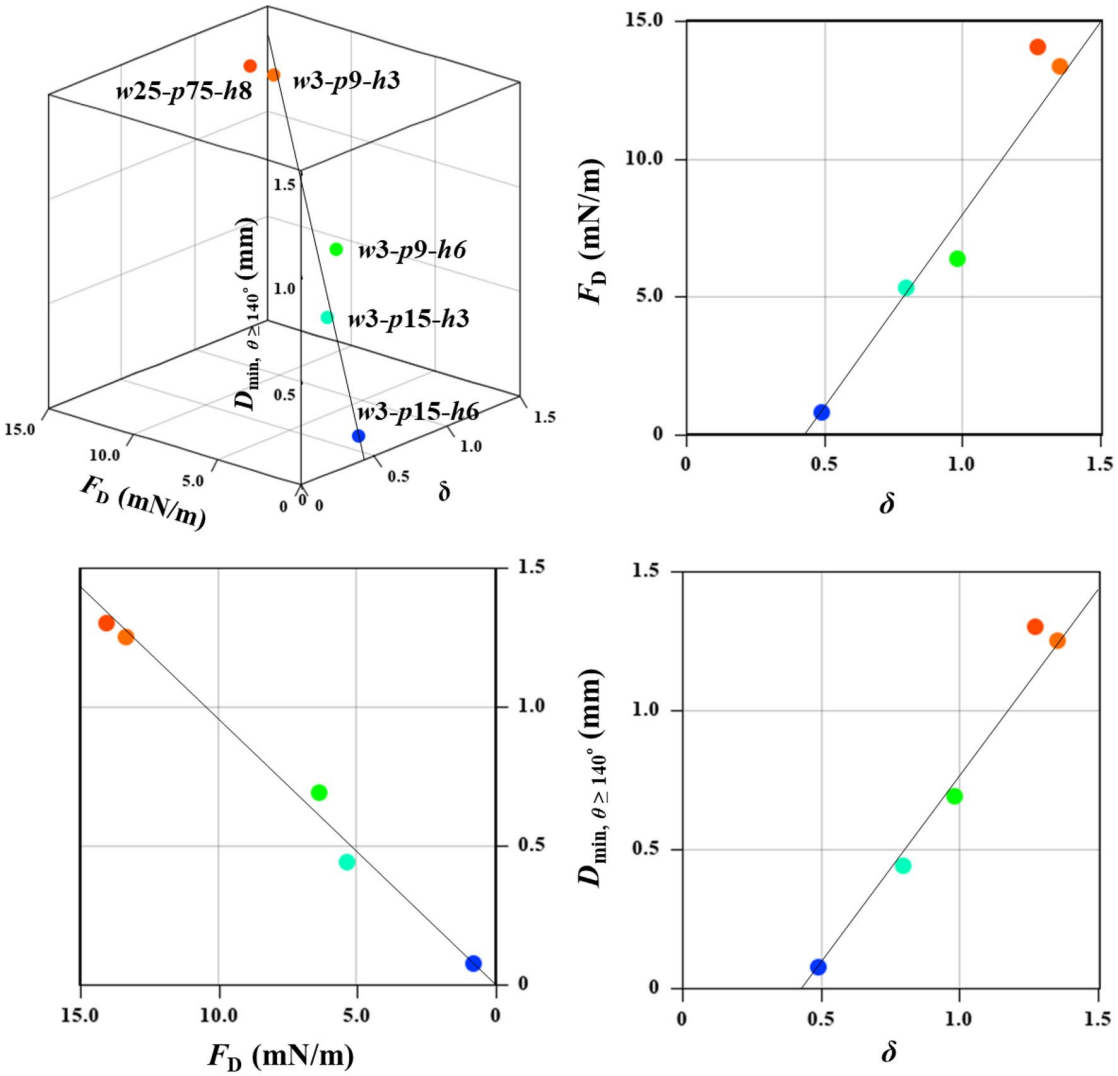
Wetting resistance (${D}_{\mathrm{min}, \theta \ge 140^\circ }$) as a function of $\delta$ and ${F}_{\mathrm{D}}$ for five different surfaces.

A similar phenomenon was observed on the *w*3-*p*9-*h*6 surface (Supplementary Fig. S3b), where the actual micropillar width also contracted to 2.29 µm from the design width of 3 µm, owing to the prolonged time for RIE (Supplementary Table S2). However, the pitch of this surface was smaller than that of the *w*3-*p*15-*h*3 surface, resulting in the third-best performance. Although the actual micropillar width of the *w*3-*p*15-*h*3 surface was larger than that of the *w*3-*p*9-*h*6 because of the shorter time for RIE, the solid fraction on the *w*3-*p*15-*h*3 was smaller than that on the *w*3-*p*9-*h*6 surface because of the larger pitch. As a result, it exhibited the second-best performance in maintaining a high CA. Thus, the current experiments revealed that designing a slippery SHS with significantly low ${F}_{\mathrm{D}}$ would significantly enhance the stability of the CB state. Reducing the solid–liquid contact by choosing the appropriate pillar pitch and employing tapered micropillars with tiny pillar width at the top edge would lead to the low ${F}_{\mathrm{D}}$ of the surface. In a study assessing the anti-fogging behavior of various sizes and shapes of nanopillars, Lecointre et al.^62^ also reported that condensed microdroplets on conical nanopillars (truncated or acute cone) exhibit higher CA and can remain highly non-wetting than on straight nanopillars. Figure 10 shows the relationship between $\delta$, ${F}_{\mathrm{D}}$, and the minimal droplet diameter that could maintain $\theta \ge 140^\circ$ (${D}_{\mathrm{min}, \theta \ge 140^\circ }$) for the five surfaces. Evidently, as $\delta$ and ${F}_{\mathrm{D}}$ decreased, ${D}_{\mathrm{min}, \theta \ge 140^\circ }$ also decreased remarkably.

## Conclusions

The wetting behavior of fakir droplets on microtextured surfaces with five different geometrical arrangements was investigated. DLC was introduced as a new substrate material, and suitable recipes were developed for cleanroom fabrication. The evaporation dynamics and wetting transition behavior of the developed surfaces were experimentally investigated. The effect of micropillar dimensions on the evaporation and transition behavior was studied. The key finding of this study is that a CB state can be maintained for micron-sized minute droplets (less than 0.37 mm in diameter) even without large pillar height or multiscale roughness when a simple slippery SHS with low depinning force was employed.

In addition, critical parameters for the Cassie–Baxter to Wenzel transition for five surfaces were estimated theoretically and experimentally, and the accuracy of the theoretical models was discussed. The results showed that the theoretical models could not precisely predict the experimental transition parameters, particularly for surfaces with short interpillar spacings. Furthermore, a numerical model was proposed and validated to predict the penetration of the suspended meniscus of the droplet on a unit cell of micropillars. The authors anticipate that the outcomes of this study will be helpful to advance various engineering applications of droplet evaporation on SHSs.

## Supplementary Information

Supplementary Legends.

Supplementary Information 1.

Supplementary Video 1.

Supplementary Video 2.

## Data Availability

The image processing code in MATLAB, as well as the script in the surface evolver for the numerical calculation of droplet meniscus penetration, is available from the corresponding author for academic purposes upon reasonable request.
