# Supplementary material for: Suppression of wetting transition on evaporative fakir droplets by using slippery superhydrophobic surfaces with low depinning force
Source: Sci Rep. 2023 Feb 9;13:2368. doi: 10.1038/s41598-023-29163-1 (PMC9911698; doi:10.1038/s41598-023-29163-1)
Supplement: Supplementary file 1 — Supplementary Legends. [file 41598_2023_29163_MOESM1_ESM.pdf]

Supplementary Information

Suppression of wetting transition on evaporative fakir droplets by using slippery superhydrophobic surfaces with low depinning force

Jubair A. Shamim,[1] Yukinari Takahashi,[1] Anjan Goswami,[2] Nadeem Shaukat,[3] Wei-Lun Hsu,[1] Junho Choi,[1] and Hirofumi Daiguji*[1]

[1]Department of Mechanical Engineering, The University of Tokyo, 7-3-1 Hongo, Bunkyo-ku, Tokyo 113-8656, Japan.

[2]Department of Mechanical Engineering, Imperial College London, London SW7 2AZ, UK.

[3]Center for Mathematical Sciences, Pakistan Institute of Engineering and Applied Sciences, Nilore 45650, Islamabad, Pakistan.

E-mail: daiguji@thml.t.u-tokyo.ac.jp

Contents

- Supplementary Note 1: Details for the fabrication of DLC-based SHS
- Supplementary Note 2: Details for the characterization of DLC-based SHS
- Supplementary Note 3: Durability test data of DLC-based SHS
- Supplementary Note 4: Details of the wetting transition experiment
- Supplementary Note 5: Parameters to evaluate the droplet dynamics on micropillars
- Supplementary Note 6: Governing equations to predict the equilibrium shape of the droplet bottom meniscus within micropillars
- Supplementary Note 7: Suppression of wetting transition on slippery SHS
- Supplementary references

Supplementary Note 1: Details for the fabrication of DLC-based SHS

The diamond-like carbon (DLC)-based superhydrophobic surface (SHS) used in this study was fabricated in a cleanroom facility at the University of Tokyo, Japan. The major fabrication steps are illustrated in Fig. 3. In addition, several intermediate cleaning steps (acetone, ethanol, and ultrasonic bath) were performed, as necessary. First, a DLC thin film (thickness: ~10 µm) was deposited on a Si wafer using the plasma-based ion implantation and deposition (PBII&D) method. Details of the DLC deposition conditions using a bipolar PBII system are available elsewhere.[1] An Al layer with a thickness of ~100 nm was deposited as a hard mask using the sputtering method (ULVAC SIH-450). Next, lithography was performed using an electron beam (EB) writer (ADVANTEST EB F5112) to transfer the pattern. OAP and ZEP520A were used as the EB resists, and ZND-50 was used as the developer.

Following the EB lithography, two reactive ion etching (RIE) steps were performed. Cl_2 plasma (ULVAC NE-550) and O_2 plasma (ULVAC CE-300I) were used to etch the Al hard mask and the DLC layer, respectively. Appropriate recipes were developed and the etching time was adjusted to obtain the desired pillar height. The conditions for RIE using O_2 plasma are listed in Supplementary Table S1. Upon the creation of the pillars, the hard mask was removed in the wet etching step using a commercial Al etchant. Finally, a Teflon (C_4F_8) layer with a thickness of ~30 nm was deposited on the micropillars using the plasma polymerization method (ULVAC MUC-21).

Supplementary Table S1 Conditions for RIE by O_2 plasma in DLC layer

Sl.	Parameters	Unit	Value
1	APC Press Set	Pa	1.0
2	Flow Set O_2	sccm	20.0
3	Antenna RF Power	W	300
4	Bias RF Power	W	100
5	Etching rate	µm/min	~0.375

Supplementary Note 2: Details for the characterization of DLC-based SHS

Five different DLC-based surfaces with different micropillar geometries were fabricated in this study. The surfaces were designated *w*3-*p*9-*h*3, *w*3-*p*9-*h*6, *w*3-*p*15-*h*3, *w*3-*p*15-*h*6, and *w*25-*p*75-*h*8 according to the design width (*w*), pitch (*p*) (center-to-center distance), and height (*h*) (Supplementary Fig. S1) of the micropillars (i.e., the specified dimensions in the layout file during the lithography step). The actual width, pitch, and height of the micropillars measured using planar and vertical scanning electron microscopy (SEM) varied slightly from the design size during fabrication. the design and actual dimensions are listed in Supplementary Table S2; 45-degree tilted and vertical SEM images of the five surfaces are shown in Supplementary Figs. S2 and S3, respectively.

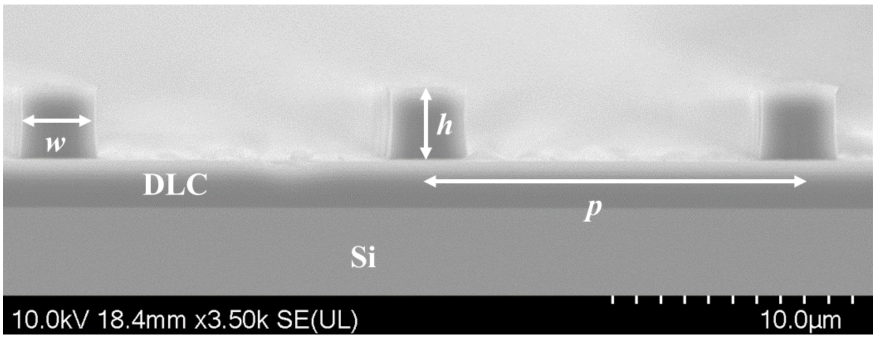

Supplementary Figure S1 Illustration of the symbols *w*, *p,* and *h* used to designate surfaces in the main text, where *w*, *p*, and *h* represent the width, pitch, and height of the pillars, respectively.

Supplementary Table S2 Micropillar dimensions of different surfaces

Sample designation	width (*w*) /µm		pitch (*p*) /µm		height (*h*) /µm	
	Design	Actual	Design	Actual	Design	Actual
*w*3-*p*9-*h*3	3.00	3.12	9.00	9.25	3.00	2.93
*w*3-*p*9-*h*6	3.00	2.29	9.00	9.29	6.00	6.80
*w*3-*p*15-*h*3	3.00	3.10	15.00	15.44	3.00	3.03
*w*3-*p*15-*h*6	3.00	1.87	15.00	15.22	6.00	6.72
*w*25-*p*75-*h*8	25.00	24.20	75.00	76.03	8.00	8.53

Note: Actual dimensions after fabrication are measured by SEM

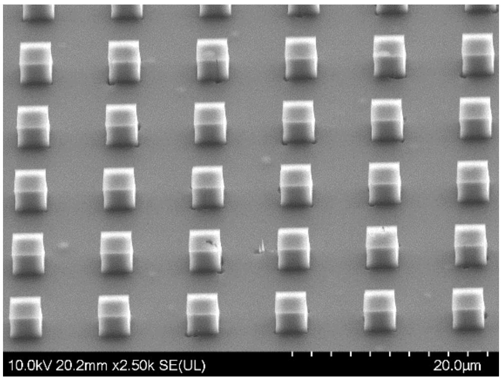

(a) *w*3-*p*9-*h*3

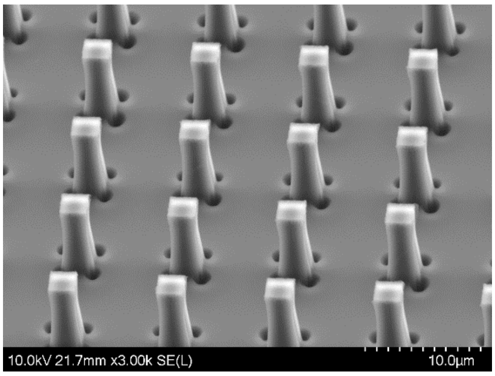

(b) *w*3-*p*9-*h*6

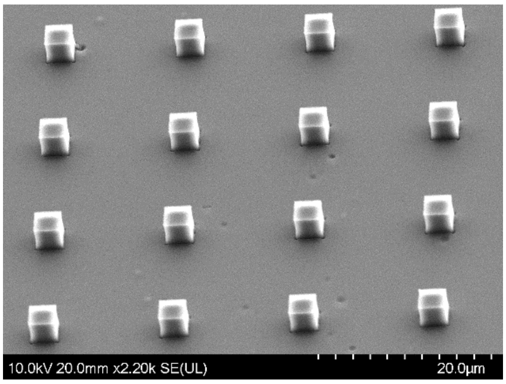

(c) *w*3-*p*15-*h*3

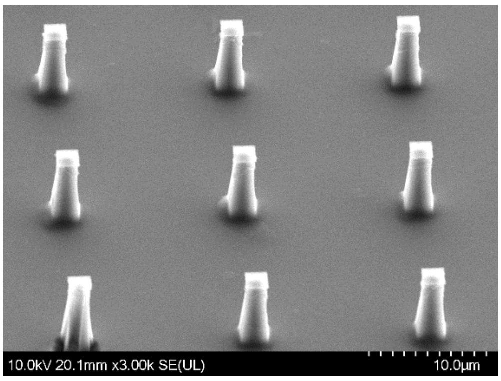

(d) *w*3-*p*15-*h*6

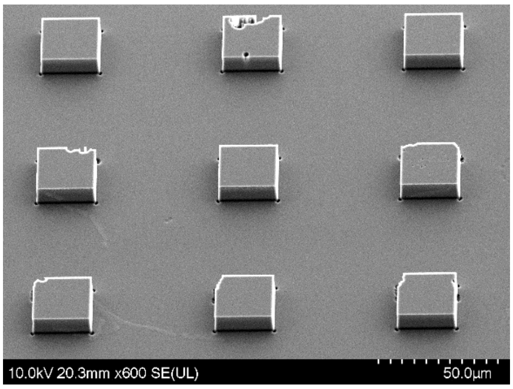

(e) *w*25-*p*75-*h*8

Supplementary Figure S2 45-degree SEM images of the micropillar array of the five surfaces in this study.

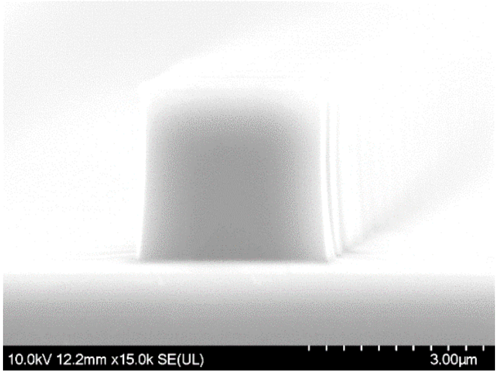

(a) *w*3-*p*9-*h*3

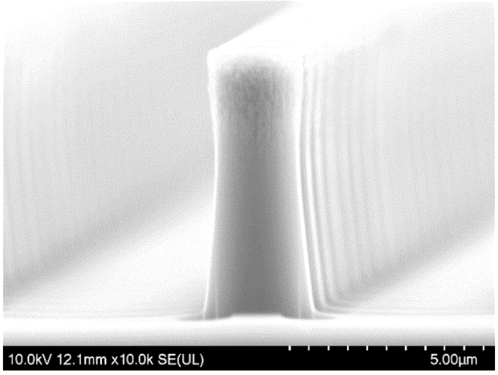

(b) *w*3-*p*9-*h*6

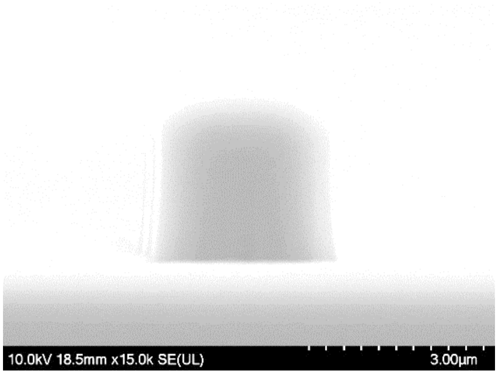

(c) *w*3-*p*15-*h*3

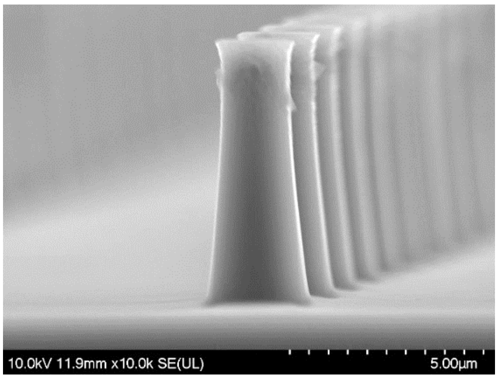

(d) *w*3-*p*15-*h*6

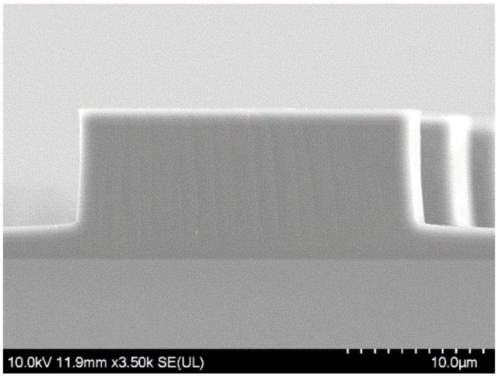

(e) *w*25-*p*75-*h*8

Supplementary Figure S3 Vertical SEM images of the five surfaces in this study.

The surfaces were also characterized in terms of static CA, roll-off angle (ROA), advancing CA, receding CA, and contact angle hysteresis (CAH). These angles were measured using a commercial CA measuring device DMo-501 (Kyowa Interface, Japan). The droplet volumes for the static and roll-off angle measurements were 2.25±0.67 μL and 2.47±0.41 μL, respectively. Small microliter droplets were generated using a syringe pump (Harvard Apparatus) and needle (Hamilton REF. 90031) connected to a Teflon tube. The tangent method was used to measure the CA and the tilting plate method (at a speed of 0.5 °/s) was used to determine the ROA. The measurements were recorded using the commercial image processing software FAMAS 5.5 provided with DMo-501. All measurements were repeated 3–5 times to determine the uncertainty of the data. The measurement results are presented in Supplementary Table S3.

Supplementary Table S3 Static CA, ROA, advancing CA, receding CA, and CAH for five surfaces

Sample designation	Static CA /μm	ROA /μm	Advancing CA /μm	Receding CA /μm	CAH /μm
*w*3-*p*9-*h*3	156.40±0.52	23.70±2.22	164.72±1.15	136.98±2.52	27.74±2.26
*w*3-*p*9-*h*6	158.63±1.21	13.33±1.15	166.73±0.80	147.50±1.38	19.23±1.65
*w*3-*p*15-*h*3	161.12±0.80	10.16±2.08	165.73±1.15	150.76±1.56	14.96±2.25
*w*3-*p*15-*h*6	157.66±1.17	4.00±0.50	164.83±1.15	156.03±0.15	8.80±1.30
*w*25-*p*75-*h*8	158.14±2.21	22.00±1.41	165.95±0.63	137.15±2.33	28.80±1.69

Supplementary Note 3: Durability test data of DLC-based SHS

The durability of the plasma deposited Teflon coating, and DLC micropillars were assessed on the *w*3-*p*9-*h*6 surface (actual pillar width, pitch, and height, respectively, 2.29 μm, 9.29 μm, and 6.80 μm) under the following three conditions: (a) thermal stability at surface temperature 105 ºC, (b) chemical inertness in 98% H_2SO_4 (Wako Pure Chemical Industries, Ltd., Japan), and (c) abrasion resistance against a #1000 grit sandpaper under 2.18 kPa stress. A total of five heating cycles were carried out (cycle duration 20 minutes), and data were measured after cooling the surface to room temperature at the end of each cycle. Similarly, for chemical inertness, the surface was immersed in 98% H_2SO_4 for a duration of 20 minutes in each cycle (up to 5 cycles), and data were measured after rinsing the surface with deionized water. Supplementary Fig. S4 shows the results of thermal stability and chemical inertness, and Supplementary Fig. S5 shows the results of abrasion resistance. The static CA and ROA remained almost the same for the repeated heating cycles (Supplementary Figs. S4(a) and (b)), and CAH increased by approximately three degrees after the first heating cycle and remained almost the same afterward (Supplementary Fig. S4(c)). Thus, the Teflon coating showed very good stability at high temperatures. Regarding the chemical inertness test, although static CA (Supplementary Fig. S4(a)) did not decrease much with successive immersion in the concentrated H_2SO_4, a significant increase in the ROA and CAH (Supplementary Figs. S4(b) and (c)) was observed with increasing the immersion time. At the end of the fifth cycle, it was confirmed in optical microscopy (Supplementary Figs. S6(a) and (b)) that the coating and pillars were not affected by the strong H_2SO_4 environment, and the increment of ROA and CAH were resulted in owing to the dirt accumulation on the surface.

Regarding the abrasion resistance test, as shown in Supplementary Fig. S5(a), static CA remained almost the same until the first 14 cm abrasion distance (initial distance 2 cm, and then at an interval of 4 cm), then increased after 20 cm (interval 6 cm), and finally lost its superhydrophobicity (due to the micropillar breakdown) after 30 cm (interval 10 cm). The ROA and CAH significantly decreased after 20 cm abrasion (Supplementary Figs. S5(b) and (c)), indicating improvement of water repellency with abrasion. The ROA and CAH after 30 cm abrasion are not reported, as the surface lost its superhydrophobicity at that distance. The increased roughness of DLC micropillars resulted in the increment of static CA and reduction in ROA and CAH until the 20 cm abrasion.

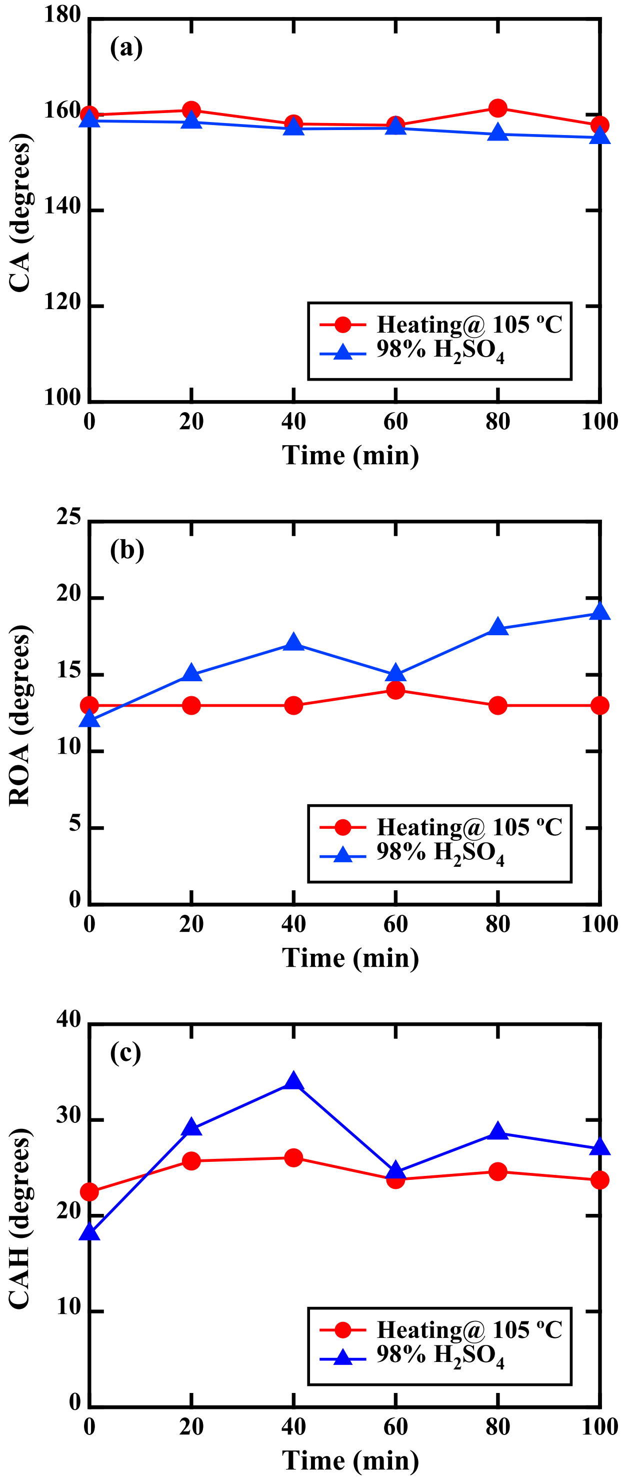

Supplementary Figure S4 Plot of (a) static CA, (b) ROA, and (c) CAH during the thermal stability and chemical inertness assessment of the *w*3-*p*9-*h*6 surface.

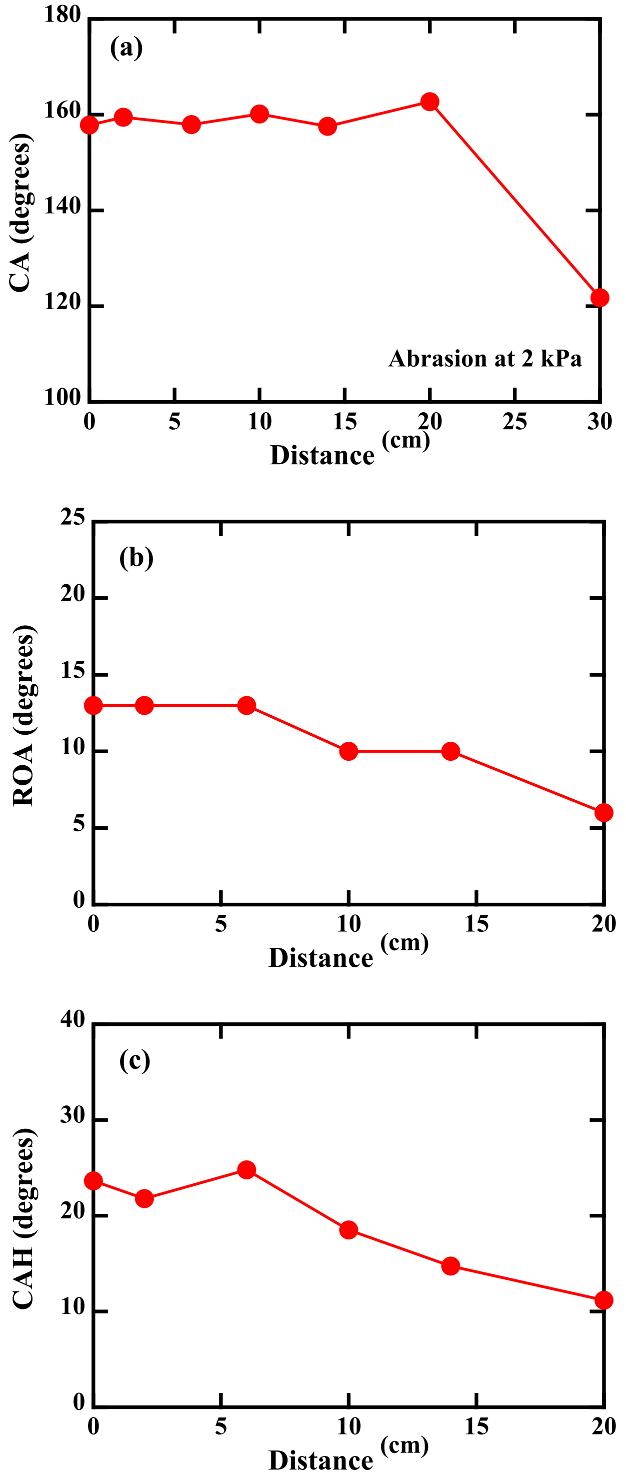

Supplementary Figure S5 Plot of (a) static CA, (b) ROA, and (c) CAH during the abrasion resistance assessment of the *w*3-*p*9-*h*6 surface.

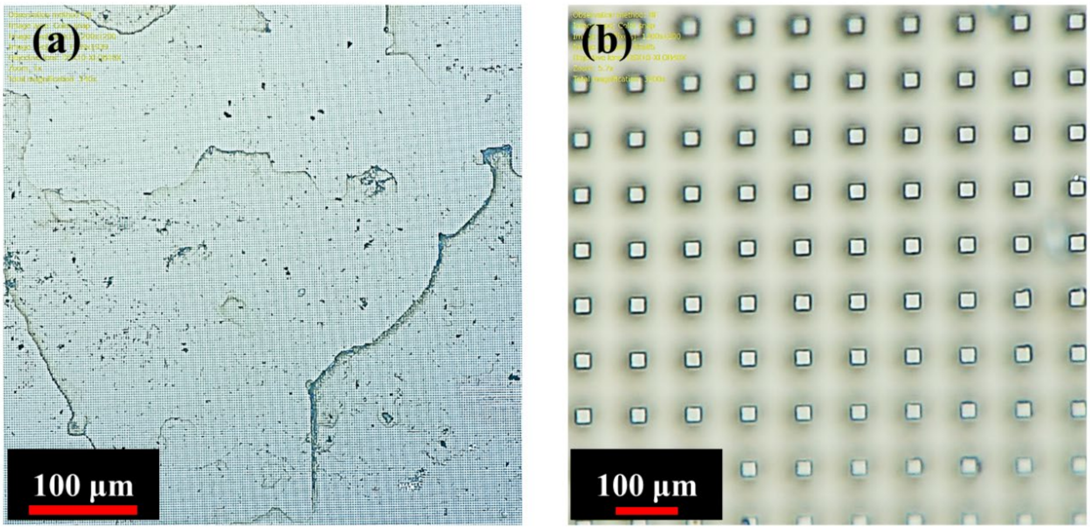

Supplementary Figure S6 (a) Dirt accumulation and (b) unaffected Teflon coating and micropillars at the end of chemical inertness test in 98% H_2SO_4.

Supplementary Note 4: Details of the wetting transition experiment

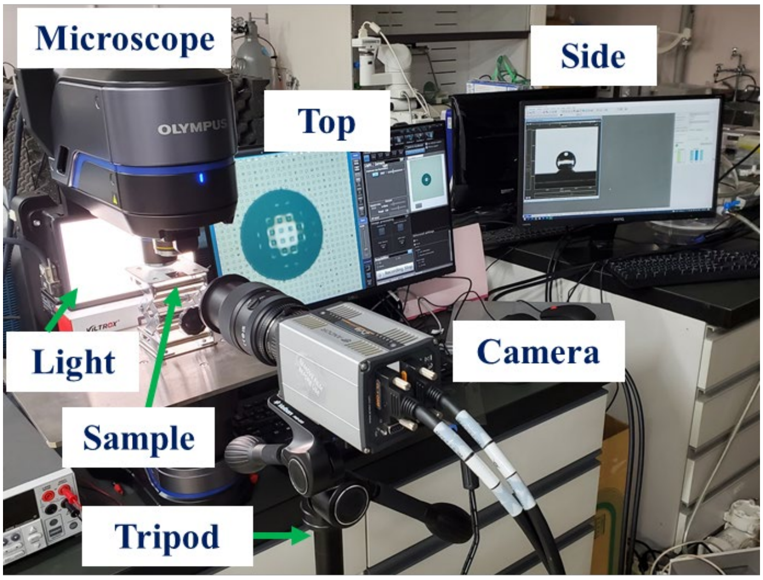

Supplementary Figure S7 Real view of the wetting transition experiment facility at the University of Tokyo.

Supplementary Table S4 Initial droplet diameter and volume at the beginning of the wetting experiment

Sample designation	Droplet diameter / mm	Droplet volume / μL
*w*3-*p*9-*h*3	1.48	1.69
*w*3-*p*9-*h*6	1.73	2.70
*w*3-*p*15-*h*3	1.58	2.06
*w*3-*p*15-*h*6	1.98	4.06
*w*25-*p*75-*h*8	1.58	2.06

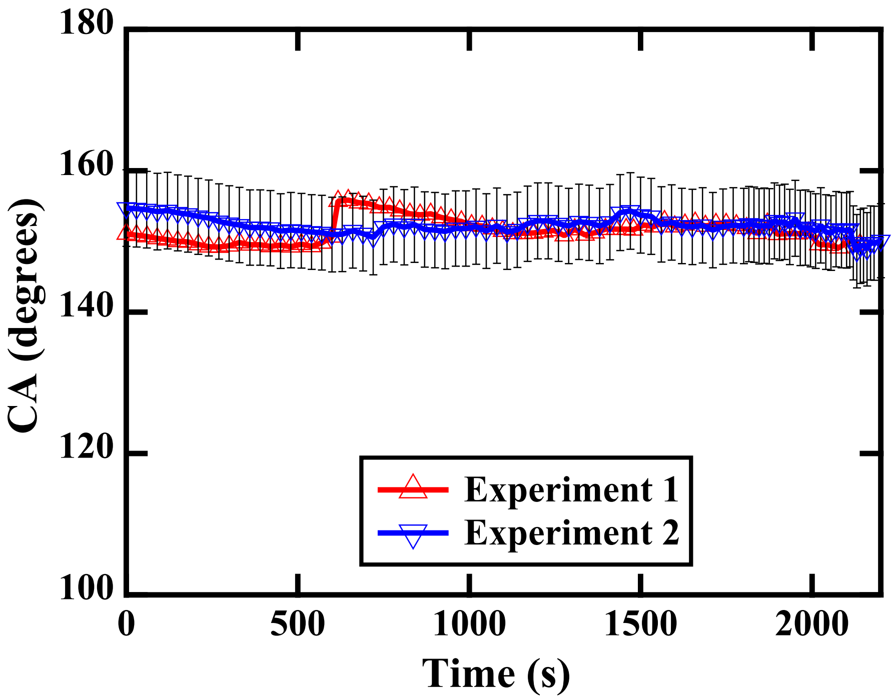

Supplementary Figure S8 Uncertainty of experimental results obtained with *w*3-*p*15-*h*6 surface.

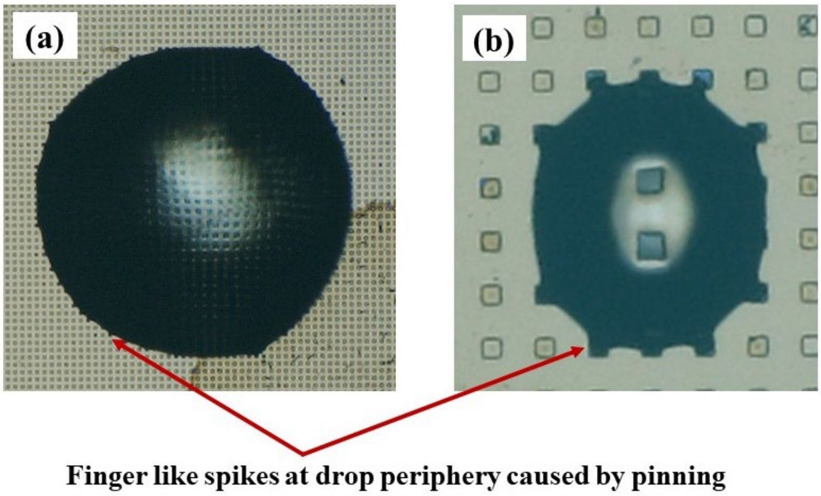

Supplementary Figure S9 Formation of the finger-like spike (top view) when the three-phase contact line is permanently pinned with the micropillars at the end stage of evaporation on the (a) *w*3-*p*9-*h*3 and (b) *w*25-*p*75-*h*8 surfaces. (Owing to the large pillar width, spikes are more clearly visible on the *w*25-*p*75-*h*8 surface.)

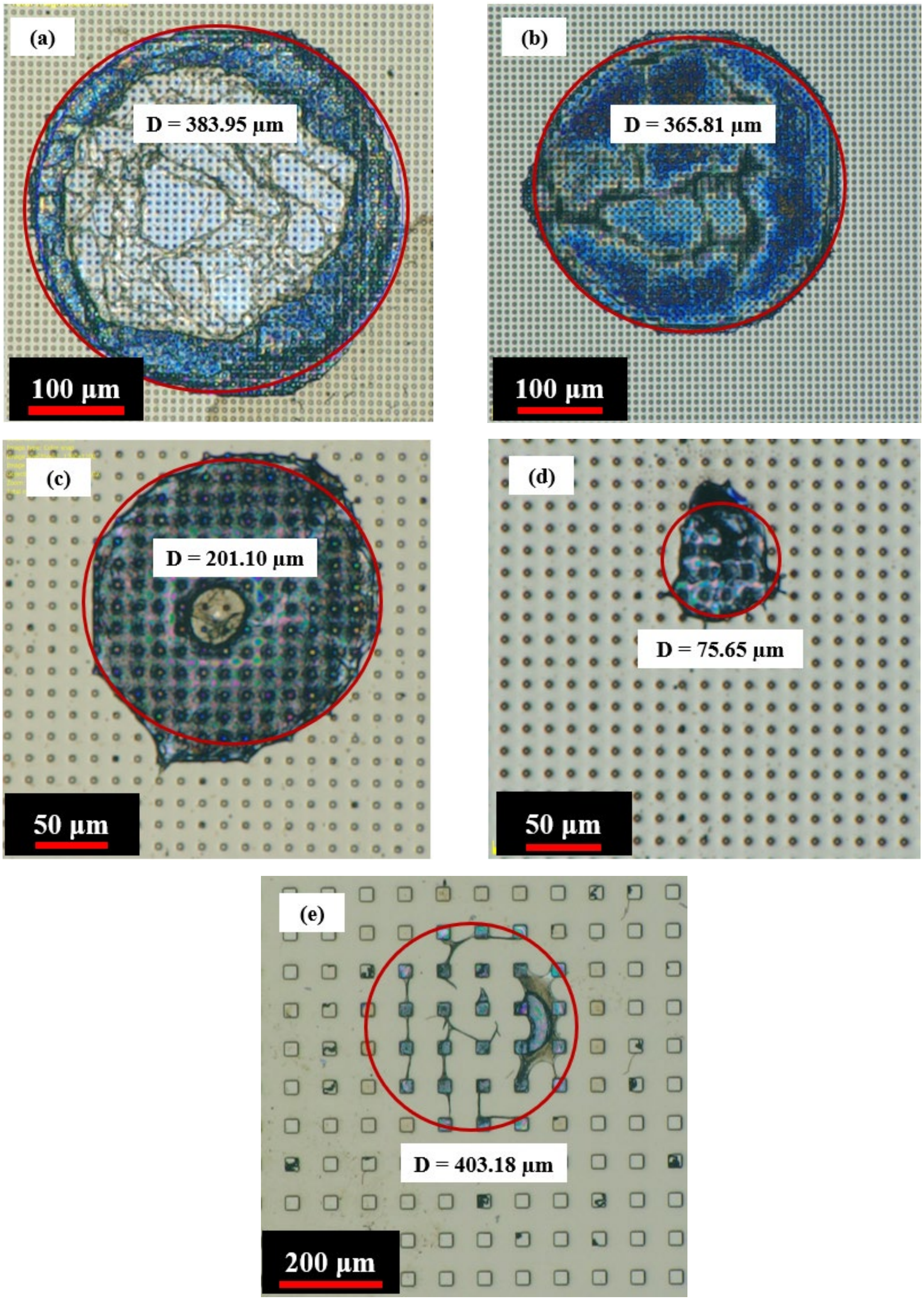

Supplementary Figure S10 Comparison of the wetted area (top) on the five surfaces after complete evaporation: (a) surface *w*3-*p*9-*h*3; (b) surface *w*3-*p*9-*h*6; (c) surface *w*3-*p*15-*h*3; (d) surface *w*3-*p*15-*h*6 and (e) surface *w*25-*p*75-*h*8.

Supplementary Note 5: Parameters to evaluate the droplet dynamics on micropillars

The solid fraction (f_s),[2] roughness factor (r),[2] normalized three-phase contact line[3] (δ), and depinning force (F_D) (i.e., force required by the three-phase contact line to overcome the solid-liquid interaction with the micropillars)[3] of each surface were calculated with actual pillar dimensions according to the following equations, and the values are tabulated in Supplementary Table S5.

$$f_\mathrm{s} = \frac{w^2}{p^2} \tag{S1}$$

$$r = 1 + \frac{4hf_\mathrm{s}}{w} \tag{S2}$$

$$\delta = \frac{4w}{p} \tag{S3}$$

$$F_\mathrm{D} = \sigma_\mathrm{lg}\,(\cos\theta_\mathrm{R} - \cos\theta_\mathrm{e}) \tag{S4}$$

In the above equations, $w, p,$ and h are the actual pillar width, pitch, and height, σ_lg is the surface tension at the liquid–gas interface (72 mN/m for water-air), θ_R is the receding CA and θ_e is the initial quasi-equilibrium CA. Here, we take the value of θ_e as the initial static CA, as reported in Supplementary Table S3, according to a previous study.[3] Supplementary Fig. S11(a) shows a graphical representation of F_D, θ_R and θ_e and Supplementary Fig. S11(b) illustrates the sticky, slippery, and super-slippery surfaces according to the normalized maximal three-phase contact line.

Supplementary Table S5 Solid fraction, roughness factor, normalized three-phase contact line, and depinning force for the five surfaces

Sample designation	Design solid fraction, $f_{\mathrm{s,d}}$ (%)	Actual solid fraction, $f_{\mathrm{s,a}}$ (%)	Roughness factor, r	Normalized three-phase contact line, δ	Depinning force, F_D (mN/m)
w3-p9-h3	11.11	11.41	1.42	1.35	13.33
w3-p9-h6	11.11	6.08	1.72	0.98	6.32
w3-p15-h3	4.00	4.03	1.15	0.80	5.30
w3-p15-h6	4.00	1.51	1.21	0.49	0.80
w25-p75-h8	11.11	10.13	1.14	1.27	14.03

Note: $f_{\mathrm{s,d}}$ and $f_{\mathrm{s,a}}$ were calculated from Supplementary Table S2 based on the design and actual pillar dimensions, respectively.

(a)

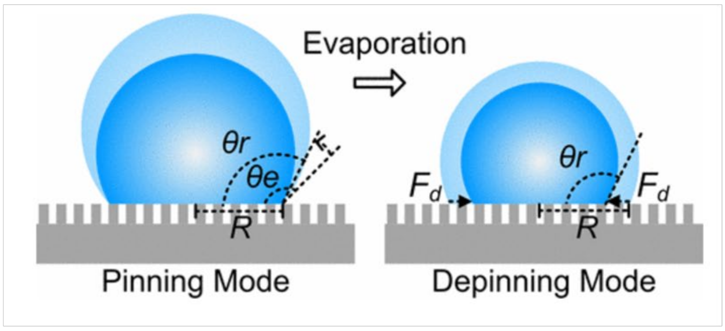

(b)

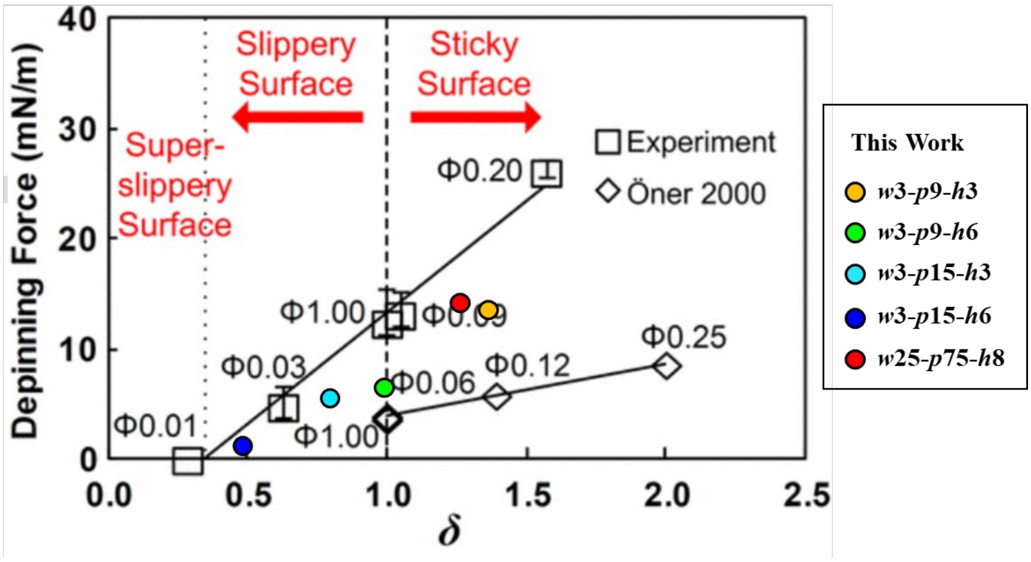

Supplementary Figure S11 (a) Graphical representation of F_D, θ_R and θ_e and (b) illustration of the sticky, slippery, and super-slippery surfaces according to the normalized maximal three-phase contact line (reprinted with permission from Supplementary reference 3).

Supplementary Note 6: Governing equations to predict the equilibrium shape of the droplet bottom meniscus within micropillars

The SE determines the final equilibrium shape of a surface subjected to different forces and constraints through energy minimization using a gradient descent method. The total energy of the droplet and the energy gradient can be expressed as follows:[4]

$$E = \iint_{A_{\mathrm{lg}}} \sigma_{\mathrm{lg}}\, dA + \iint_{A_{\mathrm{ls}}} -\sigma_{\mathrm{lg}} \cos\theta_{\mathrm{Y}}\, dA + \int\iint_{V} \rho_{\mathrm{l}}.g.z\, dV \quad \text{(S5)}$$

$$\vec{F} = -\nabla E = -(\frac{\partial E}{\partial x}\vec{\imath} + \frac{\partial E}{\partial y}\vec{\jmath} + \frac{\partial E}{\partial z}\vec{k}) \quad \text{(S6)}$$

where σ_{lg} is the surface tension of the liquid-air interface (0.072 N/m for the water–air interface), θ_{Y} is the equilibrium CA of the solid-liquid pair (110° for the water–Teflon pair), and $\vec{\imath}$, $\vec{\jmath}$, $\vec{k}$ are the unit basis vectors. In Eq. (S5), the first and second terms represent the surface tension energies at the liquid–gas and liquid–solid interfaces, respectively, and the third term denotes the effect of gravity. However, gravity is neglected in the current model because the critical droplet size at the transition is much smaller than the capillary length of the water. In Eq. (S6), $\vec{F}$ is the force applied to each node.

The liquid was assumed to completely wet the top face of the pillars, constraining the solid–liquid–air contact line at the top edges of the pillars. The wetted top faces of the pillars (i.e., the solid–liquid contact faces) were omitted, as recommended by Brakke.[5] The energy of an omitted face was assigned to the corresponding pillar edges (i.e., to the triple-phase contact line). This was achieved by transforming the area energy integral into a line integral.[5-7] Assuming $\vec{n}$ is normal to the omitted face and T is the contact energy density, we can obtain a vector field $\vec{e}$ for the line integral can be obtained as

$$\iint_{\mathrm{face}} T\vec{k}.d\vec{n} = \int_{\mathrm{edge}} \vec{e}.d\vec{l} \quad \text{(S7)}$$

where $T = -\sigma_{\mathrm{lg}} \cos\theta_{\mathrm{Y}}$. Thus, either $\vec{e} = -Ty\vec{\imath}$ or $\vec{e} = Tx\vec{\jmath}$ was used for the line integrals along the pinned three-phase contact line.

In addition to the geometric and wetting constraints, the pressure of the liquid against air (i.e., Laplace pressure) was imposed on the SE model according to the following equation:[8]

$$\Delta P_{\mathrm{Laplace}} = 2\sigma_{\mathrm{lg}}C \quad \text{(S8)}$$

where $\Delta P_{\text{Laplace}}$ is the Laplace pressure and C is the curvature of the liquid-air interface taken from the lateral view of droplets recorded at different time frames during the experiment.

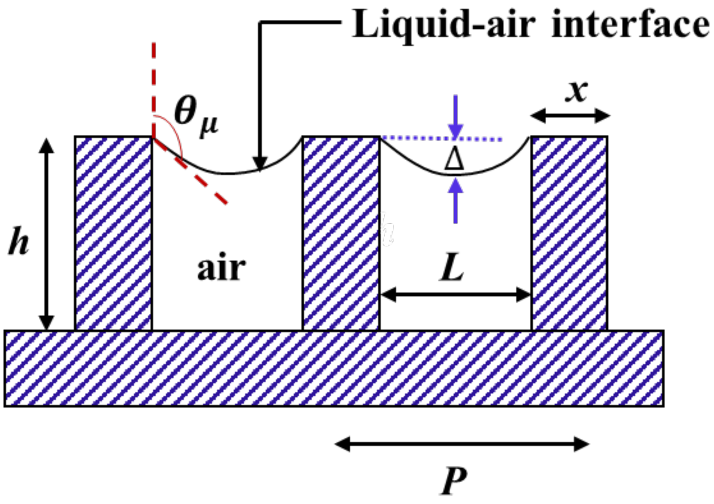

Supplementary Figure S12 Schematic representation of the droplet bottom meniscus.

Supplementary Note 7: Suppression of wetting transition on slippery SHS

According to F. Wang and H. Wu's work on the molecular origin of contact line stick-slip motion during droplet evaporation,[9] forces acting on three-phase contact line (TPCL) are: the liquid-liquid interaction force (F_{L}) which pulls the contact line towards the interior of the droplet; and (ii) the solid-liquid interaction force (F_{S}) which works to make the contact line stay on the substrate. F_{L} includes two components, F_1 acting along the liquid-vapor interface and F_2 acting along the solid-liquid interface. Supplementary Fig. S13(a) shows the graphical representation of these forces.

As the pillar width is reduced and the pitch is increased, solid-liquid contact is significantly reduced. As a result, F_{S} becomes considerably small. Therefore, in slippery SHS, the force required for depinning of the TPCL (F_{D} in Supplementary Fig. S13(a)) becomes very small during evaporation. As a result, moving TPCLs appear and continue to move to the interior micropillars (in the direction of the arrow in Supplementary Fig. S13(b)).

On the other hand, according to the theory illustrated in Fig. 2, the TPCL must remain pinned to the micropillar to maintain the CB state. On surfaces with shorter interpillar distances, at some point, θ_{μ} decreases and becomes equal to θ_{A}, and a transition occurs due to "depinning" impalement of liquid, while on surfaces with large interpillar distances, the transition mechanism changes to "sagging." If the TPCL moves at high speed on a slippery surface and the force required to overcome the solid-liquid interaction is small, the large curvature of the suspended meniscus can be maintained, and thus a large upward force can be maintained. This is the mechanism of wetting transition suppression on slippery SHS.

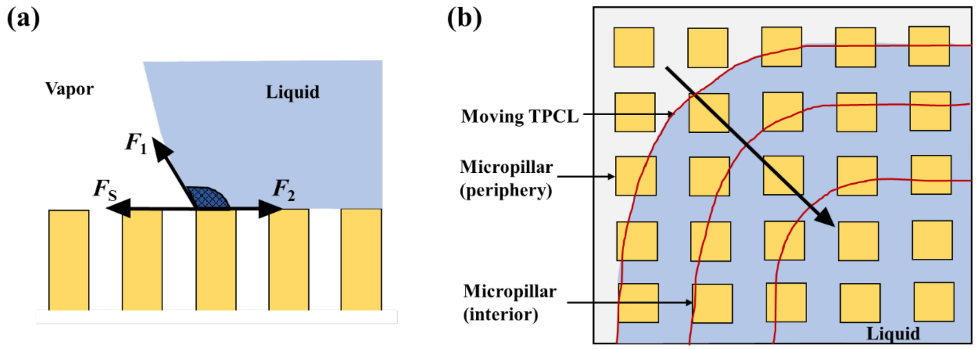

Supplementary Figure S13 (a) Representation of forces acting on TPCL (adopted with permission from Supplementary reference 9) and (b) schematic of moving TPCL.

Supplementary references

1 Choi, J., Soejima, K., Kato, T., Kawaguchi, M. & Lee, W. Nitriding of high speed steel by bipolar PBII for improvement in adhesion strength of DLC films. *Nuclear Instruments and Methods in Physics Research Section B: Beam Interactions with Materials and Atoms* **272**, 357-360, doi:https://doi.org/10.1016/j.nimb.2011.01.100 (2012).

2 Zhu, L., Feng, Y., Ye, X. & Zhou, Z. Tuning wettability and getting superhydrophobic surface by controlling surface roughness with well-designed microstructures. *Sensors and Actuators A: Physical* **130**, 595-600, doi:https://doi.org/10.1016/j.sna.2005.12.005 (2006).

3 Xu, W. & Choi, C.-H. From sticky to slippery droplets: dynamics of contact line depinning on superhydrophobic surfaces. *Physical Review Letters* **109**, 024504, doi:https://doi.org/10.1103/PhysRevLett.109.024504 (2012).

4 Elzaabalawy, A. & Meguid, S. A. Effect of surface topology on the wettability of superhydrophobic surfaces. *Journal of Dispersion Science and Technology* **41**, 470-478, doi:https://doi.org/10.1080/01932691.2019.1587299 (2020).

5 Brakke, K. A. The surface evolver. *Experimental Mathematics* **1**, 141-165, doi:https://doi.org/10.1080/10586458.1992.10504253 (1992).

6 Goswami, A., Alen, S. K., Farhat, N. & Rahman, M. Numerical study of wetting stability and sliding behavior of liquid droplets on microgrooved surfaces. *Colloid and Polymer Science* **297**, 989-1000, doi:https://doi.org/10.1007/s00396-019-04527-0 (2019).

7 Chen, Y., He, B., Lee, J. & Patankar, N. A. Anisotropy in the wetting of rough surfaces. *Journal of Colloid and Interface Science* **281**, 458-464, doi:https://doi.org/10.1016/j.jcis.2004.07.038 (2005).

8 Choi, J. *et al.* Flexible and robust superomniphobic surfaces created by localized photofluidization of azopolymer pillars. *ACS nano* **11**, 7821-7828, doi:https://doi.org/10.1021/acsnano.7b01783 (2017).

9 Wang, F. & Wu, H. Molecular origin of contact line stick-slip motion during droplet evaporation. *Scientific Reports* **5**, 1-10, doi: https://doi.org/10.1038/srep17521 (2015).
