# Supplementary material for: Suppression of wetting transition on evaporative fakir droplets by using slippery superhydrophobic surfaces with low depinning force
Source: Sci Rep. 2023 Feb 9;13:2368. doi: 10.1038/s41598-023-29163-1 (PMC9911698; doi:10.1038/s41598-023-29163-1)
Supplement: Supplementary file 2 — Supplementary Information 1. [file 41598_2023_29163_MOESM2_ESM.docx]

**Electronic supplementary material**

**Supplementary Information**

Details of the fabrication of DLC-based SHS, details of the characterization of DLC-based SHS, details of the wetting transition experiment, parameters to evaluate the droplet dynamics on micropillars, governing equations to predict the equilibrium shape of the droplet bottom meniscus within the micropillars, and explanation of transition delay due to moving TPCL are available in the supplementary material.

**Supplementary Video 1**

Permanent pinning of TPCL and completion of droplet evaporation on *w*3-*p*9-*h*3 surface from top view and lateral view.

**Supplementary Video 2**

Transition behavior of droplets and contact angle vs. droplet diameter curves on *w*3-*p*9-*h*3 and *w*3-*p*15-*h*6 surfaces (lateral view).
